# Association between Chagas disease and the occurrence of stroke: systematic review with meta-analysis

**DOI:** 10.1590/0102-311XEN246125

**Published:** 2026-08-31

**Authors:** Ana Clara de Jesus Santos, Juciane Fagundes Durães Benitez, Nayara Ragi Baldoni, Ariela Mota Ferreira, Jose Luiz Padilha da Silva, Maria do Carmo Pereira Nunes, Antonio Luiz Pinho Ribeiro, Ester Cerdeira Sabino, Desirée Sant’Ana Haikal

**Affiliations:** 1 Programa de Pós-graduação em Ciências da Saúde, Universidade Estadual de Montes Claros, Montes Claros, Brasil.; 2 Universidade de Itaúna, Itaúna, Brasil.; 3 Departamento de Estatística, Universidade Federal do Paraná, Curitiba, Brasil.; 4 Universidade Federal de Minas Gerais, Belo Horizonte, Brasil.; 5 Universidade de São Paulo, São Paulo, Brasil.

**Keywords:** Chagas Disease, Stroke, Chagas Cardiomyopathy, Heart Failure, Systematic Review, Doença de Chagas, Acidente Vascular Cerebral, Cardiomiopatia Chagásica, Insuficiência Cardíaca, Revisão Sistemática, Enfermedad de Chagas, Accidente Cerebrovascular, Cardiomiopatía Chagásica, Insuficiencia Cardíaca, Revisión Sistemática

## Abstract

This study aimed to conduct a systematic review of the association between Chagas disease and stroke, and to estimate the magnitude of this association through meta-analyses. A systematic review was conducted following the steps recommended by the *Preferred Reporting Items for Systematic Reviews and Meta-Analyses* (PRISMA) guideline. The MEDLINE via PubMed, Virtual Health Library, Web of Science, and Embase databases were searched, and all studies on the topic were considered, with no restrictions on language or publication period. Studies lacking the groups of interest (individuals with and without Chagas disease) or stroke as an outcome were excluded. Design-specific meta-analyses were performed using a random-effects model. Of the 1,695 studies identified, 7 were included in the systematic review and 6 in the meta-analysis. Among cohort studies (n = 2), the pooled meta-analysis estimate showed an increased risk of stroke among individuals with Chagas disease (RR = 1.92; 95%CI: 1.20-3.06). Among cross-sectional studies (n = 4), the pooled estimate revealed a higher prevalence of stroke among individuals with Chagas disease (PR = 1.63; 95%CI: 1.22-2.18). The certainty of evidence was rated as very low according to the GRADE approach. The results indicated an association between Chagas disease and stroke; however, they should be interpreted with caution, considering the heterogeneity across study designs and the small number of studies. Further studies with more homogeneous designs are needed to better understand this relationship.

## Abstract (pt)

Este estudo realizou uma revisão sistemática da associação entre doença de Chagas e acidente vascular cerebral (AVC), estimando a magnitude dessa associação por metanálises. Uma revisão sistemática foi realizada seguindo os passos recomendados pela *Preferred Reporting Items for Systematic Reviews and Meta-Analyses* (PRISMA) e pesquisando-se os bancos de dados MEDLINE via PubMed, Biblioteca Virtual de Saúde, Web of Science e Embase. Todos os estudos sobre o tema foram considerados, sem restrições de idioma ou período de publicação. Estudos que não incluíram os grupos de interesse (com/sem doença de Chagas) com AVC como desfecho foram excluídos. Metanálises específicas ao desenho dessa revisão foram realizadas usando um modelo de efeitos aleatórios. Dos 1.695 estudos identificados, sete foram incluídos na revisão sistemática e seis na metanálise. Entre os estudos de coorte (n = 2), a estimativa combinada de metanálise mostrou um risco aumentado de AVC entre indivíduos com doença de Chagas (RR = 1,92; IC95%: 1,20-3,06). Entre os estudos transversais (n = 4), a estimativa combinada revelou uma prevalência maior de AVC entre indivíduos com doença de Chagas (PR = 1,63; IC95%: 1,22-2,18). A certeza da evidência foi avaliada como muito baixa segundo a abordagem GRADE. Os resultados indicaram uma associação entre doença de Chagas e AVC. No entanto, eles devem ser interpretados com cautela dado o pequeno número de estudos e sua heterogeneidade. São necessários estudos adicionais com desenhos mais homogêneos para entender melhor essa relação.

## Abstract (es)

Este estudio realizó una revisión sistemática de la asociación entre la enfermedad de Chagas (EC) y el accidente cerebrovascular (AVC), que estimó la magnitud de esta asociación a través de metaanálisis. Se realizó una revisión sistemática siguiendo los pasos que recomienda la *Preferred Reporting Items for Systematic Reviews and Meta-Analyses* (PRISMA) y realizando búsquedas en las bases de datos MEDLINE a través de PubMed, Biblioteca Virtual en Salud, Web of Science y Embase. Se consideraron todos los estudios sobre el tema, sin restricciones de idioma ni período de publicación. Se excluyeron los estudios que no incluían a los grupos de interés (con/sin enfermedad de Chagas) con el AVC como resultado. Los metaanálisis específicos para el diseño de esta revisión se realizaron utilizando un modelo de efectos aleatorios. De los 1.695 estudios identificados, siete se incluyeron en la revisión sistemática y seis en el metaanálisis. Entre los estudios de cohorte (n = 2), la estimación combinada del metaanálisis mostró un aumento del riesgo de AVC entre las personas con enfermedad de Chagas (RR = 1,92; IC95%: 1,20-3,06). Entre los estudios transversales (n = 4), la estimación combinada demostró una mayor prevalencia de AVC entre las personas con enfermedad de Chagas (PR = 1,63; IC95%: 1,22-2,18). La certeza de la evidencia se evaluó como muy baja según la metodología GRADE. Los resultados indicaron una asociación entre la enfermedad de Chagas y el AVC. Sin embargo, dichos resultados deben interpretarse con prudencia debido al pequeño número de estudios y a su heterogeneidad. Se necesitan más estudios con diseños más homogéneos para mejor comprender esta relación.

## Introduction

Chagas disease remains an important public health problem in large parts of Latin America ^1^. The chronic phase of Chagas disease entails not only a risk of death but also multiple physical, cardiac, digestive, immunological, and neurological sequelae ^1^. Among the mechanisms associated with Chagas disease, ischemic cerebrovascular events stand out, particularly stroke ^2^. Stroke is a highly prevalent neurological condition, a relevant cause of temporary or permanent disability, and a major public health problem ^3^.

Some studies indicate a higher occurrence of stroke among individuals with Chagas disease ^4^ ^,^ ^5^. Chagas disease has been identified as one of the main cardiomyopathies associated with the development of stroke, resulting in severe sequelae, unfavorable prognosis, and an increased risk of long-term mortality ^5^ ^,^ ^6^ ^,^ ^7^ ^,^ ^8^. Risk factors for stroke in patients with Chagas disease include apical aneurysm, left ventricular thrombus, significant atrial dilation, left ventricular systolic dysfunction, advanced age, and atrial arrhythmias ^9^.

Although the association between Chagas disease and stroke has been described in the literature, controversies and lack of clarity persist. This relationship has been addressed by isolated studies that adopted different study designs, different measures of association, reported varying magnitudes of association, and not all results were concordant. Thus, to provide greater clarity on this topic, the present study aims to conduct a systematic review of the literature on the association between Chagas disease and stroke, as well as to estimate the magnitude of this association through meta-analyses conducted according to different study designs.

## Methods

### Study design

A systematic review was conducted following the steps recommended by the *Preferred Reporting Items for Systematic Reviews and Meta-Analyses* (PRISMA) guideline ^10^. The study protocol was registered in the International Prospective Register of Systematic Reviews (PROSPERO; identification number CRD42023488516). The research question originally registered was: “Do individuals with Chagas disease have a higher risk of stroke compared with individuals without the disease?”. Due to the scarcity of longitudinal studies addressing this topic, the research question was expanded to “Is there an association between Chagas disease and the occurrence of stroke in adults?”, allowing the inclusion of observational studies with different designs.

### Study eligibility criteria

The search was developed based on the question formulated using the PECOS strategy: Population: adults; Exposure: Chagas disease; Comparison: without Chagas disease; Outcome: stroke; and Study design: observational studies (cohort, case-control, and cross-sectional), analyzed separately. Given the limited literature on the topic and the intention to produce a robust body of evidence, studies with different observational designs were included.

**Figure 1:**
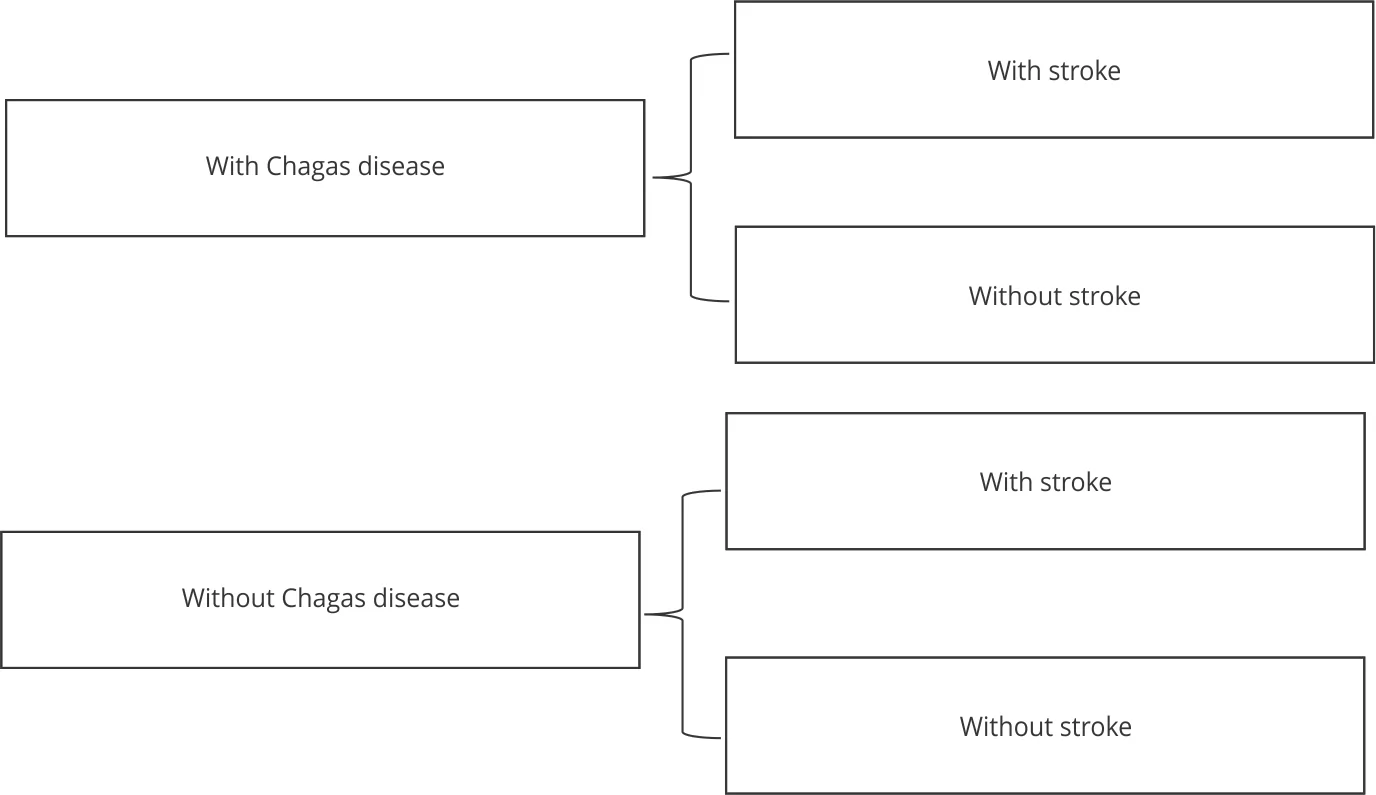
Study eligibility criteria.

Thus, observational studies conducted in adult populations that evaluated the association between Chagas disease and the occurrence of stroke were considered eligible. Chagas disease was defined according to the diagnostic criteria adopted by the included studies themselves (serology, clinical criteria, and/or laboratory criteria). The outcome of interest was the occurrence of stroke, including ischemic and hemorrhagic stroke, according to the definitions used in the original studies. Only studies providing sufficient information to identify four distinct groups (Figure 1) were considered eligible. No restrictions were applied regarding language, publication period, or sample origin (endemic or non-endemic regions). Review articles, descriptive studies, protocols, editorials, letters to the editor, news items, and commentaries were excluded.

### Resources used in the study

The following databases were searched to identify relevant studies: MEDLINE via PubMed, Virtual Health Library, Web of Science, and Embase. In addition, a search of the gray literature was conducted to reduce publication bias. A manual search of the reference lists of the included studies and of systematic reviews on the topic was also performed to identify potentially eligible additional studies. Searches were conducted in April 2024 and updated in January 2026.

### Search strategy

The descriptors adopted in the search strategy were identified in the lists provided by Medical Subject Headings (MeSH Terms) and Health Sciences Descriptors (DeCS; acronym in Portuguese). Searches were performed using combinations of the terms “Chagas disease” and “stroke”. Box 1 presents the exact terms used in the searches. The complete search strategy for each database is available in Supplementary Material (https://cadernos.ensp.fiocruz.br/static//arquivo/suppl-e00246125_7851.pdf).

**Box 1:** MEDLINE (via PubMed) search strategy.

(“Chagas Disease”ENT#091;MeSH TermsENT#093; OR “American Trypanosomiasis”ENT#091;All FieldsENT#093; OR “trypanosomiasis american”ENT#091;All FieldsENT#093; OR “trypanosomiasis south american”ENT#091;All FieldsENT#093; OR “South American Trypanosomiasis”ENT#091;All FieldsENT#093; OR “Trypanosoma cruzi Infection”ENT#091;All FieldsENT#093; OR “infection trypanosoma cruzi”ENT#091;All FieldsENT#093; OR “infections trypanosoma cruzi”ENT#091;All FieldsENT#093; OR “Trypanosoma cruzi Infections”ENT#091;All FieldsENT#093; OR “Chagas Disease”ENT#091;All FieldsENT#093;) AND (“Stroke”ENT#091;MeSH TermsENT#093; OR (“Stroke”ENT#091;MeSH TermsENT#093; OR “Stroke”ENT#091;All FieldsENT#093; OR “strokes”ENT#091;All FieldsENT#093; OR “stroke s”ENT#091;All FieldsENT#093;) OR “cerebrovascular accident*”ENT#091;All FieldsENT#093; OR “cva cerebrovascular accident”ENT#091;All FieldsENT#093; OR “Cerebrovascular Apoplexy”ENT#091;All FieldsENT#093; OR “apoplexy cerebrovascular”ENT#091;All FieldsENT#093; OR “vascular accident brain”ENT#091;All FieldsENT#093; OR “brain vascular accident*”ENT#091;All FieldsENT#093; OR “cerebrovascular stroke*”ENT#091;All FieldsENT#093; OR “stroke* cerebrovascular”ENT#091;All FieldsENT#093; OR (“apoplexies”ENT#091;All FieldsENT#093; OR “Stroke”ENT#091;MeSH TermsENT#093; OR “Stroke”ENT#091;All FieldsENT#093; OR “apoplexy”ENT#091;All FieldsENT#093;) OR “cerebral stroke*”ENT#091;All FieldsENT#093; OR “stroke* cerebral”ENT#091;All FieldsENT#093; OR “stroke* acute”ENT#091;All FieldsENT#093; OR “acute stroke*”ENT#091;All FieldsENT#093; OR “cerebrovascular accident* acute”ENT#091;All FieldsENT#093; OR “acute cerebrovascular accident*”ENT#091;All FieldsENT#093; OR “ischemic stroke”ENT#091;All FieldsENT#093; OR “Brain Infarction”ENT#091;MeSH TermsENT#093; OR “brain infarction*”ENT#091;All FieldsENT#093; OR “infarction* brain”ENT#091;All FieldsENT#093; OR “brain infarct*”ENT#091;All FieldsENT#093; OR “infarct* brain”ENT#091;All FieldsENT#093; OR “venous infarction brain”ENT#091;All FieldsENT#093; OR “brain venous infarction*”ENT#091;All FieldsENT#093; OR “venous infarctions brain”ENT#091;All FieldsENT#093; OR “venous brain infarction*”ENT#091;All FieldsENT#093; OR “Anterior Cerebral Circulation Infarction”ENT#091;All FieldsENT#093; OR “Anterior Circulation Brain Infarction”ENT#091;All FieldsENT#093; OR “brain infarction posterior circulation”ENT#091;All FieldsENT#093; OR “Posterior Circulation Brain Infarction”ENT#091;All FieldsENT#093; OR “Embolic Stroke”ENT#091;MeSH TermsENT#093; OR “Embolic Strokes”ENT#091;All FieldsENT#093; OR “stroke* embolic”ENT#091;All FieldsENT#093; OR “cardioembolic stroke*”ENT#091;All FieldsENT#093; OR “stroke* cardioembolic”ENT#091;All FieldsENT#093; OR “cardio embolic stroke*”ENT#091;All FieldsENT#093; OR “Cardio embolic Stroke”ENT#091;All FieldsENT#093; OR “stroke* cardio embolic”ENT#091;All FieldsENT#093;)

The selected articles were exported to the Rayyan web platform (https://www.rayyan.ai/) ^11^, which uses machine-learning algorithms to identify potential duplicate records. This resource was used to flag duplicates, without automatic exclusion. Duplicate records were carefully verified manually by the researchers before the final decision on exclusion, ensuring human oversight at all stages.

### Study selection

Study selection and data extraction using a standardized form were performed independently by two researchers (A.C.J.S. and J.F.D.B.). Disagreements were resolved by two additional reviewers (N.R.B. and D.S.H.). Studies were selected in three phases: (1) rapid screening of titles and abstracts, (2) careful reading of abstracts, and (3) full-text reading.

In the first phase, articles containing both search terms, or their respective synonyms, in the title and/or abstract were selected, as presented in Box 1. These studies proceeded to the second phase.

In the second phase, titles and abstracts were read in full. Studies were considered eligible for the next stage if the abstracts provided sufficient information on the study population (groups with Chagas disease and without Chagas disease) and the outcome of interest (occurrence of stroke). Abstracts that were incomplete or did not clearly allow determination of eligibility were also fully read.

In the third phase, full texts were read to confirm compliance with the inclusion criteria. Among the studies selected at this stage, two were identified as abstracts published in conference proceedings. In these cases, the authors were contacted to verify the existence of a full publication and/or to obtain additional information on the results and methodology used.

Studies that did not present the groups of interest (Figure 1) defined in the eligibility criteria were excluded. In cases of different publications derived from the same sample, only the most complete version was considered. The reasons for exclusion of studies assessed at the full-text stage are detailed in the Supplementary Material (https://cadernos.ensp.fiocruz.br/static//arquivo/suppl-e00246125_7851.pdf).

### Database creation process

The following data were extracted from the studies selected after the third phase: study design, authors, year of publication, sample size, stroke prevalence/incidence, and effect measure. The information was organized in a Microsoft Office Excel 2010 spreadsheet (https://products.office.com/).

### Risk of bias and methodological quality

The risk of bias of the included studies was assessed independently by two reviewers (A.C.J.S. and J.F.D.B.) using design-specific instruments. For cohort and case-control studies, the *Risk Of Bias In Non-randomized Studies of Exposures* (ROBINS-E) tool ^12^ was used, which was developed to assess observational epidemiological studies in the context of systematic reviews. ROBINS-E allows a systematic appraisal of the robustness of evidence regarding the existence and magnitude of the effect of an exposure on an outcome, based on the explicit definition of the estimated causal effect in each study. The instrument comprises seven bias domains, assessed through signaling questions that inform judgment of the risk of bias in each domain, considering the potential impact of bias on the effect estimate and its relevance for interpretation of the results. At the end of this assessment, an overall risk-of-bias judgment was assigned to each study, following the hierarchical approach recommended by the instrument, in which the overall risk reflects the highest risk identified among the assessed domains and is classified as low risk, unclear risk, or high risk of bias. Details of the application of the ROBINS-E tool, including domain-level judgments for each study, are available in the Supplementary Material (https://cadernos.ensp.fiocruz.br/static//arquivo/suppl-e00246125_7851.pdf).

The risk of bias of cross-sectional studies was assessed using the JBI tool ^13^, which provides a structured approach to evaluating the methodological quality of this design. The instrument allows systematic appraisal of aspects related to sample selection, measurement of exposure and outcome, identification and control of confounding factors, and adequacy of statistical analyses. The tool consists of a set of questions answered as “yes”, “no”, “unclear”, or “not applicable”, based on which an overall judgment of the methodological quality of each study was made, classified as high, moderate, or low, considering the set of responses and the potential impact of bias on interpretation of the results. Details of the application of the JBI tool, including responses to each item and the overall judgment for each study, are available in the Supplementary Material (https://cadernos.ensp.fiocruz.br/static//arquivo/suppl-e00246125_7851.pdf).

### Assessment of the certainty of evidence

The certainty of evidence of this review was assessed using the *Grading of Recommendations Assessment, Development, and Evaluation* (GRADE) approach ^14^. The domains considered for downgrading the certainty of evidence were risk of bias, inconsistency, indirectness, imprecision, and publication bias. Conversely, criteria that may increase the certainty of evidence were also evaluated, including magnitude of effect, dose-response gradient, and the possibility of adjustment for confounding factors. Based on the assessment of these domains, the certainty of evidence was classified as high (≥ 4 points), moderate (3 points), low (2 points), or very low (1 point).

### Meta-analysis

Meta-analyses were conducted separately according to the different study designs, respecting the need for greater standardization and homogeneity of the groups. The identified differences were described.

Meta-analyses were performed using a random-effects model, with estimates presented together with 95% confidence intervals (95%CI). For cross-sectional studies, effects were expressed as prevalence ratios (PR), calculated as the ratio between event proportions. The type of complication was considered in the subgroup analysis. For longitudinal (cohort) studies, results were presented as relative risk (RR).

Between-study variance (τ^2^) was estimated using the restricted maximum likelihood method (REML). Heterogeneity was assessed using the I^2^ statistic, which quantifies the proportion of total variability attributable to heterogeneity between studies, and the τ² parameter. The statistical significance of heterogeneity was examined using Cochran’s Q test, considering the corresponding p-value. Analyses were performed using R software, version 4.4.2 (http://www.r-project.org), with the meta package. The link to access the database and the complete script used to conduct the meta-analyses are available in the Supplementary Material (https://cadernos.ensp.fiocruz.br/static//arquivo/suppl-e00246125_7851.pdf).

## Results

### Study selection

**Figure 2:**
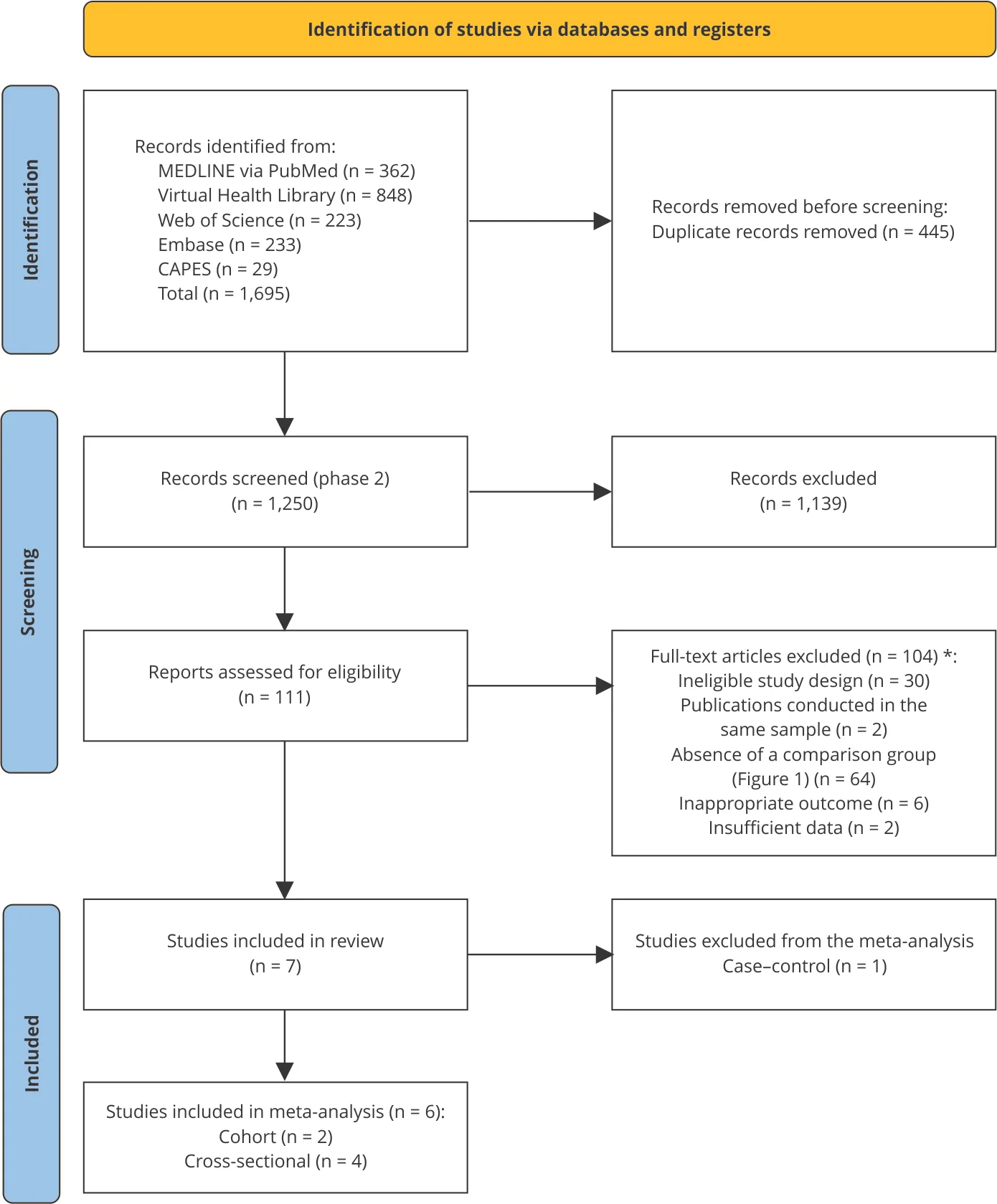
PRISMA (*Preferred Reporting Items for Systematic Reviews and Meta-Analyses*) flow diagram of the methodological process for study selection in the conduct of the systematic review.

Based on the searches conducted, 1,695 studies were identified, of which 445 were duplicates. After applying the exclusion criteria, 111 studies were read in full, of which 7 were included in the systematic review and 6 were included in the meta-analysis (Figure 2). Two abstracts published in conference proceedings were identified, and the corresponding authors were contacted. For one of the abstracts, the author responded that the study resulted in a scientific article that was subsequently published, which had already been identified and included in this review. For the other abstract, it was not possible to retrieve additional data, as no further information was available beyond that presented in the proceedings. The manual search, conducted through analysis of the reference lists of the included studies and of other reviews on the topic, did not identify additional eligible studies, as all relevant studies had already been located through the electronic searches.

* The detailed reasons for the exclusion of articles are described in the Supplementary Material (https://cadernos.ensp.fiocruz.br/static//arquivo/suppl-e00246125_7851.pdf).

### Study characteristics

Table 1 shows the characteristics of the studies included in the review, as well as the description of the comparison groups (patients with Chagas disease vs. patients without Chagas disease) and their association with stroke. All studies were conducted in Brazil, although they were published in national and international journals. Articles published between 2005 and 2021 were included. The number of participants considered in the studies ranged from 79 to 1,398. Detailed clinical characteristics (ejection fraction, arrhythmias, and thrombi) and methodological parameters (statistical adjustments, loss to follow-up, and funding) are presented in Table 2.

**Table 1:** Main characteristics, results, and assessment of risk of bias and methodological quality of the included studies (n = 7).

Design (Study)	Population/Sample and setting	Sample size	Stroke prevalence or incidence	Main findings/Effect measure (95%CI)	Risk of bias/Methodological quality
Cohort					
Cerqueira-Silva et al. ^15^ (2022)	Heart failure outpatient clinic at a university hospital. Bahia, Brazil	Total: 404 patients G1: heart failure with Chagas disease (n = 210) vs. G2: heart failure without Chagas disease (n = 194)	There were 25 cases of ischemic stroke (G1: 16 cases; G2: 9 cases)	Chagas disease was an independent predictor of first ischemic stroke (HR = 2.54; 95%CI: 1.01-6.42)	High risk of bias
Lima-Costa et al. ^16^ (2010)	Community-dwelling residents aged ≥ 60 years identified through a census. Bambuí, Brazil	Total: 1,398 participants G1: individuals with Chagas disease (n = 524) vs. G2: individuals without Chagas disease (n = 874)	There were 45 stroke-related deaths (G1: 25 cases; G2: 20 cases)	Individuals with Chagas disease had a higher risk of death from stroke compared with those without Chagas disease (HR = 2.35; 95%CI: 1.25-4.44)	High risk of bias
Cross-sectional					
Matta et al. ^18^ (2012)	Medical records of consecutive patients from a cardiomyopathy outpatient clinic. Bahia, Brazil	Total: 790 patients G1: Chagas cardiomyopathy (n = 329) vs. G2: non-Chagas cardiomyopathy (n = 461)	There were 108 cases of ischemic stroke (G1: 57 cases; G2: 51 cases)	Stroke was more frequent among patients with Chagas disease (OR = 1.79; p = 0.012)	High methodological quality
Dias et al. ^19^ (2009)	Random sample of patients from a cardiomyopathy outpatient clinic at a university hospital. Bahia, Brazil	Total: 79 patients G1: Chagas cardiomyopathy (n = 37) vs. G2: other cardiomyopathies (n = 42)	There were 12 cases of ischemic stroke (G1: 6 cases; G2: 6 cases)	Cognitive dysfunction was common among patients with Chagas disease, and a history of stroke was more prevalent in this group, although not statistically significant (p = 0.81)	High methodological quality
Jesus et al. ^21^ (2011)	Cardiomyopathy outpatient clinic of the Federal University of Bahia. Bahia, Brazil	Total: 144 patients G1: CHF with Chagas disease (n = 62) vs. G2: CHF due to other etiologies (n = 82)	There were 13 cases of stroke (G1: 7 cases; G2: 6 cases)	Stroke was more frequent among patients with Chagas disease, although the association was not statistically significant (p = 0.563)	Moderate methodological quality
Oliveira-Filho et al. ^20^ (2005)	Patients with heart disease treated at a tertiary care outpatient clinic. Bahia, Brazil	Total: 305 patients G1: patients with Chagas disease (n = 147) vs. G2: patients without Chagas disease (n = 158)	There were 32 cases of stroke (G1: 22 cases; G2: 10 cases)	Chagas disease was an independent risk factor for stroke (OR = 1.09; 95%CI: 1.02-1.17)	High methodological quality
Case-control					
Montanaro et al. ^17^ (2019)	Neurological rehabilitation service at SARAH Hospital. Brasília, Brazil	Total: 115 patients G1: AF without Chagas disease (n = 49) vs. G2: indeterminate Chagas disease (n = 23) vs. G3: cardioembolic Chagas disease (n = 43)	There were 13 stroke cases (G1: 6 cases; G2: 3 cases; G3: 4 cases)	No differences were observed in stroke mortality or recurrence between groups (p = 0.073)	High risk of bias

95%CI: 95% confidence interval; AF: atrial fibrillation; CHF: congestive heart failure; HR: hazard ratio; OR: odds ratio.

**Table 2:** Characteristics of the studies included in the review (n = 7).

Study	Population and clinical severity (Chagas group)	Operational definitions (exposure/comparator/outcome)	Inclusion/Exclusion criteria and diagnostic method	Follow-up (time and losses)	Effect measure and adjustments for confounders	Funding source
Cerqueira-Silva et al. ^15^ (2022)	LVEF: median 47%. AF: 7.8%. Apical aneurysm: 3.4%. Thrombus: not available in table	Exposure: ELISA test performed in duplicate. Comparator: non-Chagas heart failure etiologies. Outcome: ischemic stroke	Inclusion: age > 18 years, Framingham criteria for heart failure. Exclusion: previous stroke, anticoagulation, recent thrombotic event, terminal diseases, and neurodegenerative diseases. Diagnostic: questionnaire, echocardiogram, ECG, serology, CT/MRI	Time: cohort, mean 42.9 months. Losses: 145 patients lost to follow-up	HR: 2.54 (95%CI, 1.01-6.42). Adjustments: sex, age, Chagas disease, acetylsalicylic acid use, dyslipidemia, LVEF, and AF	Authors declare no financial support
Lima-Costa et al. ^16^ (2010)	LVEF: not evaluated (population-based). AF: 3.3%. Thrombus/Aneurysm: not available	Exposure: positivity in 3 assays (IHA and 2 ELISA). Comparator: Uninfected individuals. Outcome: death due to stroke (ICD-10)	Inclusion: residents aged ≥ 60 years in 1997. Exclusion: missing or inconclusive serology. Diagnostic: serology, ECG, death verification	Time: 10-year cohort (1997-2007). Losses: 6% of the original cohort	HR: 2.35 (95%CI, 1.25-4.44). Adjustments: age, sex, education, conventional risk factors, and CRP	FINEP, Brazilian Ministry of Health, and FAPEMIG
Matta et al. ^18^ (2012)	LVEF: < 45%. AF: 8.2%. Thrombus: 2.8%. Aneurysm: not available in table	Exposure: positive serology (two tests: IFA, HAI, or ELISA). Comparator: non-Chagas cardiomyopathy. Outcome: ischemic stroke (focal deficit > 24h)	Inclusion: Diagnosis of structural cardiomyopathy. Exclusion: epidural and subdural hemorrhage, poisoning, trauma, coma, and lack of information on stroke. Diagnostic: ECG, echocardiogram, serology, radiography, and other examinations	Time: cross-sectional (medical record review). Losses: 16 individuals without stroke data	OR: 1.79 (95%CI: 1.13-2.84). Adjustments: age, sex, hypertension, pacemaker, AF, CAD	Authors declare no support or funding
Dias et al. ^19^ (2009)	LVEF: mean 37.4%. AF: 13.5%. Thrombus/Aneurysm: not detailed in table, but thrombus cited as a disease marker	Exposure: serology (hemagglutination or immunofluorescence). Comparator: other cardiomyopathies. Outcome: cognitive dysfunction (MMSE and Rey Figure)	Inclusion: LVEF < 40% or 40-49% with dilated chambers. Exclusion: blindness, renal/hepatic failure, cardiac arrest, COPD, hypothyroidism. Diagnostic: serology, ECG, echocardiogram	Time: cross-sectional. Losses: 13 patients excluded after screening	OR: 4.63 for MMSE (p = 0.07) and 4.67 for Rey Figure. Adjustments: age, education, digoxin, hypertension, stroke, CAD	CAPES, NIH, and CNPq
Jesus et al. ^21^ (2011)	LVEF: mean 37.8% with Chagas disease and 36.4% without Chagas disease. AF: 3%. Pacemaker: 11%. Thrombus/Aneurysm: not available in table	Exposure: etiology defined by cardiologist and serology. Comparator: other causes of heart failure (hypertensive, ischemic, etc.). Outcome: SCM	Inclusion: patients with CHF, available echocardiogram. Exclusion: use of oral anticoagulants. Diagnostic: TCD, echocardiogram	Time: cross-sectional (TCD monitoring for 60min). Losses: not reported	OR: 1.15 (95%CI: 1.05-1.26). Adjustments: age and LVEF	Not reported
Oliveira-Filho et al. ^20^ (2005)	LVEF: 40 to 49%. AF: 4.9%. Thrombus/Aneurysm: 4%	Exposure: positive serology in two tests. Comparator: individuals with other cardiomyopathies. Outcome: stroke	Inclusion: clinical signs suggestive of cardiomyopathy. Exclusion: not reported. Diagnostic: serology, ECG, echocardiogram, questionnaire, and neurological assessment	Time: cross-sectional (conducted between 2002 and 2003). Losses: not reported	HR: 2.36 (95%CI: 1.25-4.44). Adjustments: age, sex, education, conventional risk factors, and CRP	CNPq and NIH
Montanaro et al. ^17^ (2019)	LVEF: reduced in 11 patients (25.5%). Apical aneurysm: 10.6% (7/66). Thrombus: 4.5% (3/66)	Exposure: 2wo serological tests (ELISA and HAI). Comparator: AF without Chagas disease. Outcome: vascular topography of brain lesions in Chagas disease and stroke	Inclusion: stroke confirmed by imaging, diagnosis of Chagas disease or AF. Exclusion: < 18 years, conflicting serology, lack of imaging. Diagnostic: medical records, CT/MRI, ECG, echocardiogram	Time: case-control (retrospective, patients admitted 2009-2013; followed until 2017). Losses: 20% of the population with Chagas disease	Statistics: frequency comparison (chi-square) and Kaplan-Meier (no OR/HR for group effect in the excerpts)	No specific funding

95%CI: 95% confidence interval; AF: atrial fibrillation; CAD: coronary artery disease; CAPES: Brazilian Coordination for the Improvement of Higher Education Personnel; CHF: congestive heart failure; CNPq: Brazilian National Research Council; COPD: chronic obstructive pulmonary disease; CRP: C-reative protein; CT: computed tomography; ECG: electrocardiogram; ELISA: enzyme-linked immunosorbent assay; FAPEMIG: Minas Gerais State Research Foundation; FINEP: Brazilian Funding Authority for Studies and Projects; HAI: hemagglutination inhibition assay; HR: hazard ratio; ICD-10: International Classification of Diseases (10th Revision); IFA: indirect immunofluorescence assay; LVEF: left ventricular ejection fraction; MMSE: *Mini Mental State Examination*; MRI: magnetic resonance imaging; NIH: National Institutes of Health (United States); OR: odds ratio; SCM: silent cerebral microembolism; TCD: transcranial doppler.

Regarding study design, cohort studies (n = 2), cross-sectional studies (n = 4), and a case-control study (n = 1) were identified. With respect to risk of bias assessment, the cohort ^15^ ^,^ ^16^ and case-control ^17^ studies were classified as having a high risk of bias according to the ROBINS-E tool. This classification was predominantly determined by limitations in the domains of confounding, participant selection, post-exposure interventions, and missing data (Supplementary Material; https://cadernos.ensp.fiocruz.br/static//arquivo/suppl-e00246125_7851.pdf). Among the cross-sectional studies, three showed high methodological quality ^18^ ^,^ ^19^ ^,^ ^20^ while one was considered of moderate quality ^21^ due to limitations in exposure measurement, according to the JBI tool (Supplementary Material; https://cadernos.ensp.fiocruz.br/static//arquivo/suppl-e00246125_7851.pdf).

Among the studies included in this review, differences were observed in the composition of the groups with and without Chagas disease, as some studies selected participants based on specific cardiac conditions. Among the cohort studies, one was conducted exclusively with participants with heart failure ^15^, while the other did not present information on or assess the cardiac condition of the participants ^16^. Among the cross-sectional studies, three included only patients with cardiomyopathy ^18^ ^,^ ^19^ ^,^ ^20^, while the other adopted patients with congestive heart failure (CHF) as an inclusion criterion ^21^. The case-control study was conducted with patients with Chagas disease in the indeterminate or cardioembolic forms and with patients without Chagas disease who had atrial fibrillation (AF) ^17^, as shown in Table 1.

**Figure 3:**
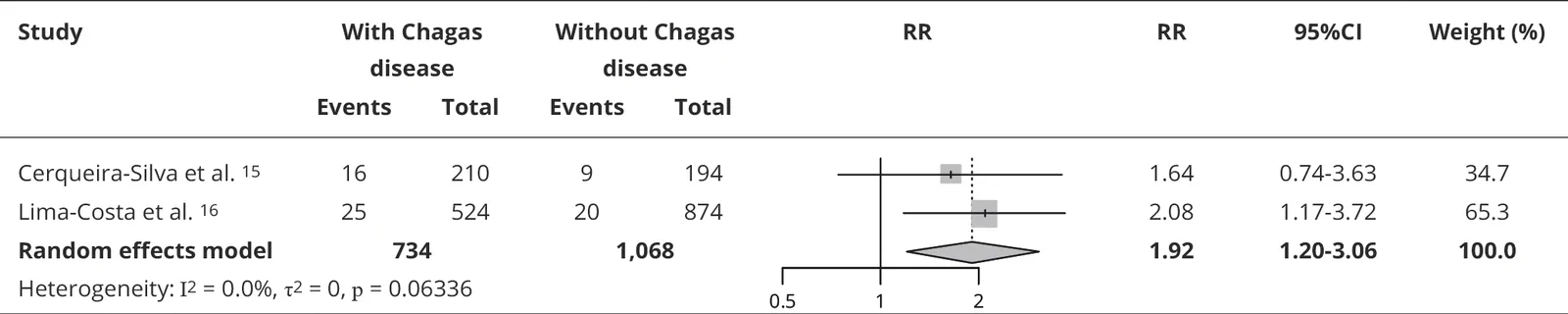
Meta-analysis of cohort studies on the association between Chagas disease and stroke. Forest plot showing relative risk (RR) and 95% confidence intervals (95%CI) for stroke in individuals with Chagas disease compared with those without Chagas disease *.

Figure 3 presents the meta-analysis of the pooled data from the two cohort studies included. In the combined estimate, an increased risk of stroke was observed among individuals with Chagas disease (RR = 1.92; 95%CI: 1.20-3.06). No statistically significant heterogeneity was observed between the studies (I^2^ = 0%; Cochran’s Q test: p > 0.05).

* Exposure: Chagas disease; Outcome: stroke; Effect measure: RR; Statistical model used: random-effects model.

**Figure 4:**
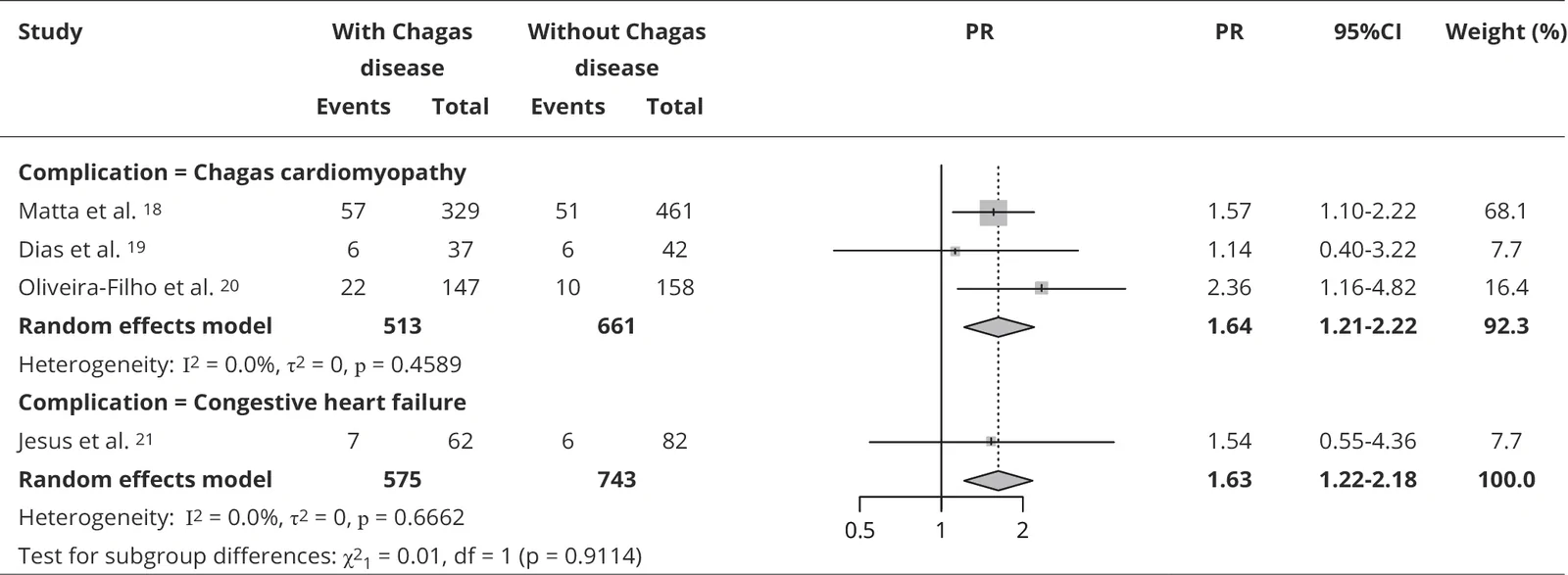
Meta-analysis of cross-sectional studies assessing the occurrence of stroke in individuals with Chagas disease compared with those without Chagas disease, according to type of cardiac complication. Forest plot showing prevalence ratios (PR) and the corresponding 95% confidence intervals (95%CI) *.

Figure 4 presents the meta-analysis of the pooled data from the cross-sectional studies. In the combined estimate, an increased prevalence of stroke was observed among individuals with Chagas disease (PR = 1.63; 95%CI: 1.22-2.18). No statistically significant difference was observed between the subgroups analyzed according to type of complication (test for differences between subgroups: p > 0.05), suggesting that the magnitude of the association was similar between Chagas cardiomyopathy and heart failure. No statistically significant heterogeneity was observed (I^2^ = 0%; Cochran’s Q test: p > 0.05).

* Exposure: Chagas disease; Outcome: stroke; Effect measure: PR; Statistical model used: random-effects model.

It was not possible to conduct a meta-analysis for case-control studies, as only one study with this design met the inclusion criteria, precluding quantitative synthesis of findings from different studies.

According to the GRADE approach, the overall certainty of evidence for the evaluated outcomes was classified as very low. Downgrading of the certainty of evidence was mainly due to methodological limitations, inconsistency across studies, and imprecision of the estimates (Table 3).

**Table 3:** Certainty of evidence for the association between Chagas disease and stroke, assessed according to the GRADE (*Grading of Recommendations Assessment, Development, and Evaluation*) approach.

Outcome and follow-up	Patients (studies)	Relative effect (95%CI)	Absolute effects (95%CI)			Certainty	What happens
			Comparison	Intervention	Difference		
Occurrence of stroke and stroke mortality	1,802 (2 cohort studies)	RR = 1.92 (95%CI: 1.20-3.06)	56 per 1.000	107 per 1,000 (67 to 171)	51 more per 1,000 (11 more to 115 more)	⨁◯◯◯ Very low * **	Individuals with Chagas disease have a higher risk of developing stroke during follow-up compared with those without the disease
Occurrence of stroke	1,318 (4 cross-sectional studies)	PR = 1.63 (95%CI: 1.22-2.18)	160 per 1.000	261 per 1,000 (195 to 349)	***	⨁◯◯◯ Very low ^#^	Individuals with Chagas disease have a higher prevalence of stroke compared with those without the disease

95%CI: 95% confidence interval; PR: prevalence ratio; RR: risk ratio.

* Both cohort studies presented a high risk of bias according to the ROBINS-E tool, mainly due to survivor bias associated with a prevalent user design, as well as substantial loss to follow-up in one of the studies;

** Downgrading for imprecision due to the limited number of studies and events, as well as the wide confidence intervals;

*** The absolute effect was not calculated by the GRADE approach for the cross-sectional studies;

^#^ The overall risk of bias was considered serious. Three studies showed high methodological quality according to the JBI checklist, whereas one study presented an unclear assessment of exposure measurement, introducing some uncertainty. However, no critical methodological flaws were identified across the studies.

## Discussion

The findings of this systematic review with meta-analysis indicated an association between Chagas disease and stroke, with a higher likelihood of presenting/developing stroke among individuals with Chagas disease compared with those without the disease. This association was observed in the meta-analyses of cohort and cross-sectional studies, as well as in the analysis of the single case-control study included in this review. Nevertheless, these results should be interpreted with caution given the small number of included studies and the geographic concentration of investigations in a single country. Clinical and methodological differences among the studies included in the meta-analyses must also be considered. Although no statistically significant heterogeneity was observed (I^2^ = 0%), suggesting consistency of the estimates, the absence of heterogeneity should not be interpreted as definitive evidence of homogeneity.

The results are consistent with the existing literature, although methodological differences explain variations in relation to previous reviews, particularly with regard to eligibility criteria. Previous systematic reviews ^22^ ^,^ ^23^ adopted broader approaches, including studies with different designs and considering both Chagas disease and stroke as the initial condition of interest. The present study used more restrictive criteria, including exclusively analytical investigations that assessed Chagas disease as the exposure, with a control group and stroke as a clearly defined outcome, in addition to requiring methodological information and results that allowed comparisons between groups and quantitative synthesis, which explains why some studies included in previous reviews were excluded.

Initially, the objective of this study was to conduct a systematic review considering only cohort studies; however, only two studies with this design were identified in the literature addressing this topic. For this reason, it was necessary to include other study designs to provide greater robustness and strengthen the evidence presented. As stroke can occur at any stage of life among individuals with Chagas disease ^24^, it is important to understand the factors contributing to stroke occurrence in order to reduce its frequency in this population. Thus, the importance of further cohort studies to better elucidate this association is emphasized. It is important to consider that cross-sectional studies do not allow causal inference and may be subject to selection or information bias, especially when data are based on secondary records.

In Chagas disease, cardiomyopathy is independently associated with ischemic stroke; however, little is known about its incidence and the factors associated with an increased risk of stroke occurrence in these individuals ^20^ ^,^ ^25^. In a cohort of patients with Chagas disease, the annual incidence of cardioembolic stroke was 0.56% during a mean follow-up of 5.5 years ^2^. A study included in this review demonstrated that the risk of death due to stroke in individuals with Chagas disease may reach 30%, corresponding to twice that observed among non-infected individuals, with stroke mortality estimated at 4.6 per 1,000 patient-years; in addition, elevated levels of brain natriuretic peptide (BNP) were predictors of stroke death among older adults with chronic Chagas disease ^16^. In a cohort study included in this review ^15^, conducted among patients with CHF, Chagas disease was a predictor of stroke and death independently of the severity of heart disease. The incidence of stroke was higher in patients with Chagas disease compared with those without Chagas disease (20.2 vs. 13.9 events per 1,000 patient-years) ^15^. These findings reinforce the hypothesis that Chagas disease represents an important independent risk factor for ischemic stroke, possibly related to mechanisms specific to Chagas cardiomyopathy.

A study conducted with 678 patients with Chagas disease identified 72 cases of stroke and indicated that heart failure, right bundle branch block, left anterior superior fascicular block, and AF were the associated causes ^26^. The etiology of stroke in Chagas disease has been described as predominantly cardioembolic, accounting for approximately 85% to 90% of cases ^24^ ^,^ ^27^ ^,^ ^28^. However, other mechanisms have been identified, including large-artery atherosclerosis and small-vessel occlusion ^29^. A narrative review presented results supporting the hypothesis that Chagas disease itself may confer a significant independent risk for stroke, potentially through mechanisms distinct from those in the general population ^23^.

In addition to classic cardioembolic mechanisms, patients with Chagas disease present a systemic pro-inflammatory and pro-thrombotic state that may contribute to stroke occurrence ^29^. Chronic infection with *Trypanosoma cruzi* is associated with persistent activation of the inflammatory response and the coagulation cascade, with potential impact on endothelial function and hemostatic balance, favoring an environment conducive to thrombosis and cerebrovascular events, even in the absence of advanced cardiomyopathy ^22^ ^,^ ^29^. A clinical study further indicated that the occurrence of stroke in individuals with Chagas disease is not restricted to patients with severe cardiac dysfunction, suggesting the involvement of additional systemic mechanisms beyond classic cardioembolism ^30^. In this context, processes such as chronic inflammation, endothelial dysfunction, and activation of the coagulation system have been described as potential pathophysiological pathways involved in the increased risk of stroke in Chagas disease, broadening the understanding of the mechanisms underlying this association ^23^. A systematic review without meta-analysis reported that stroke may occur in up to 20% of cases of chronic Chagas disease ^31^; however, it did not include articles that evaluated comparison groups without Chagas disease in which stroke outcomes were assessed.

On the other hand, an anatomopathological study conducted in an endemic area did not identify differences in the overall frequency of stroke between individuals with and without Chagas disease, although differences in the profile of stroke subtypes were observed ^32^. This finding suggests that Chagas disease may influence the etiological pattern of cerebrovascular events without necessarily increasing their overall frequency in all contexts.

Although the studies included in this review employed different designs, they demonstrated a possible association between Chagas disease and stroke. Moreover, this association has already been reported in previous studies, in which the main causes associated with stroke manifestation in patients with Chagas disease were apical aneurysm and the presence of left ventricular thrombi, conditions that are common in patients with Chagas disease ^9^. Another longitudinal study that evaluated stroke incidence among individuals with Chagas disease showed an overall incidence of 2.67 events per 100 patient-years, with reduced ejection fraction and left atrial volume identified as risk factors ^33^; however, neither of these studies included comparison groups without Chagas disease. One of the studies included in this systematic review showed that the risk of death from stroke among participants with Chagas disease was twice that observed among participants without Chagas disease ^16^. In a case-control study conducted among patients with stroke compared with patients with acute coronary syndrome, positive serology for Chagas disease was significantly more frequent in the stroke group than in the acute coronary syndrome group (p = 0.002). After adjustment in the final model, positive Chagas disease serology remained independently associated with stroke (odds ratio ENT#091;ORENT#093; = 7.17; 95%CI: 1.50-34.19) ^34^.

Preventive strategies, particularly anticoagulation in patients at high risk of cardioembolic stroke in Chagas disease, may reduce the occurrence of cerebrovascular events. A recent systematic review on anticoagulant therapy showed that this intervention was associated with a significant reduction in ischemic stroke among patients with Chagas disease (OR = 0.28; 95%CI: 0.19-0.41), highlighting its potential role in primary and secondary prevention of these events in high-risk profiles ^35^.

Regarding clinical heterogeneity, variability was observed in the population profiles evaluated, particularly with respect to the severity of cardiomyopathy and heart failure. While some studies included general populations, others predominantly assessed individuals with established Chagas cardiomyopathy at different stages of severity. This heterogeneity limited direct comparability of findings and precluded more refined stratified analyses. Such clinical variability may influence the magnitude of the observed association between Chagas disease and stroke, considering that Chagas cardiomyopathy is associated with a higher risk of cardioembolic events ^36^. It should be noted that, among the studies included in this review, no investigations addressing the association between stroke and Chagas disease in the acute phase of the disease were identified.

This systematic review has limitations that should be considered when interpreting the findings. The included studies showed heterogeneity in design, reflecting the scarcity of investigations assessing the association between stroke and Chagas disease with adequate comparison groups, which resulted in a limited number of eligible studies. In the meta-analysis, studies with differences in the definition of exposure and comparison groups were combined, including investigations that evaluated patients with heart failure with and without Chagas disease, as well as studies that included individuals with Chagas disease without prior assessment of cardiac status in the comparison groups. In addition, the meta-analysis combined studies that assessed stroke occurrence and stroke mortality, which may introduce outcome indirectness and influence effect estimates. Although no statistical heterogeneity was observed (I^2^ = 0%), this finding should be interpreted with caution given the small number of included studies (n = 7). Formal assessment of publication bias was not performed due to the insufficient number of studies to apply graphical methods or statistical tests; nonetheless, strategies were adopted to minimize this risk, such as inclusion of gray literature, searches in multiple databases, and transparency regarding potentially eligible studies that were not included. On the other hand, this review included all studies that met the eligibility criteria, regardless of design, provided that they presented the outcome of interest and a comparison group.

Despite these limitations, the findings of this study point to the potential relevance of the association between Chagas disease and stroke, although it has not yet been definitively elucidated. Therefore, in addition to conducting further studies, strategies for stroke surveillance and prevention are recommended in regions endemic for Chagas disease, with a focus on cardiological follow-up, early diagnosis, and control of associated risk factors.

## Conclusion

This systematic review with meta-analysis investigated the association between Chagas disease and stroke and identified evidence of a higher occurrence of stroke among individuals with Chagas disease. Seven studies were included (four cross-sectional, two cohort, and one case-control). The meta-analysis of cohort studies demonstrated an approximately twofold higher risk of stroke among individuals with Chagas disease compared with those without Chagas disease. In cross-sectional studies, a 63% higher prevalence of stroke was observed in this group. The single case-control study included showed convergent results, although it was not possible to conduct a meta-analysis for this design.

Interpretation of the findings should consider important limitations, such as the small number of studies, heterogeneity of study designs, clinical differences among the groups analyzed, and the concentration of investigations in a single country, which restricts the generalizability of the results.

Nevertheless, this review contributes to advancing knowledge by systematically synthesizing the available evidence on the topic and indicating an association between Chagas disease and stroke. The results reinforce the need for longitudinal studies with greater methodological standardization, as well as the importance of strategies for stroke prevention and management among people with Chagas disease, especially in endemic areas, aiming to reduce morbidity and mortality and improve quality of life.

## Data Availability

The databases used in the study, including extraction codes, analyses, and results, are available in the repository SciELO Data: https://doi.org/10.48331/SCIELODATA.VUA9MU.
