# Supplementary material for: Association between Chagas disease and the occurrence of stroke: systematic review with meta-analysis
Source: Cad Saude Publica. 2026 Aug 31;42:e00246125. doi: 10.1590/0102-311XEN246125 (PMC13539625; doi:10.1590/0102-311XEN246125)
Supplement: Supplementary file 1 [file 1678-4464-csp-42-EN246125-s.pdf]

Association between Chagas disease and the occurrence of stroke: a systematic review and meta-analysis

Summary

Supplement 1 – Full Literature Search Strategy 2
Supplement 2 – Selection and exclusion of studies 5
Supplement 3 – Risk of bias and methodological quality 30
3.1 Risk of bias assessment: (ROBINS-E) 30
3.1.1 Supplementary figure 1 51
3.2 Assessment of the methodological quality: (JBI) 52
3.2.1 Supplementary figure 2 53
Supplement 4 – Scripts 54
Supplement 5 – Database 56

Supplement 1 – Full Literature Search Strategy (Search conducted on January 13, 2025, across all databases)

Database	Search strategy
PubMed<br>**362**	("Chagas Disease"[MeSH Terms] OR "American Trypanosomiasis"[All Fields] OR "trypanosomiasis american"[All Fields] OR "trypanosomiasis south american"[All Fields] OR "South American Trypanosomiasis"[All Fields] OR "Trypanosoma cruzi Infection"[All Fields] OR "infection trypanosoma cruzi"[All Fields] OR "infections trypanosoma cruzi"[All Fields] OR "Trypanosoma cruzi Infections"[All Fields] OR "Chagas Disease"[All Fields]) AND ("Stroke"[MeSH Terms] OR ("Stroke"[MeSH Terms] OR "Stroke"[All Fields] OR "strokes"[All Fields] OR "stroke s"[All Fields]) OR "cerebrovascular accident*"[All Fields] OR "cva cerebrovascular accident"[All Fields] OR "Cerebrovascular Apoplexy"[All Fields] OR "apoplexy cerebrovascular"[All Fields] OR "vascular accident brain"[All Fields] OR "brain vascular accident*"[All Fields] OR "cerebrovascular stroke*"[All Fields] OR "stroke* cerebrovascular"[All Fields] OR ("apoplexies"[All Fields] OR "Stroke"[MeSH Terms] OR "Stroke"[All Fields] OR "apoplexy"[All Fields]) OR "cerebral stroke*"[All Fields] OR "stroke* cerebral"[All Fields] OR "stroke* acute"[All Fields] OR "acute stroke*"[All Fields] OR "cerebrovascular accident* acute"[All Fields] OR "acute cerebrovascular accident*"[All Fields] OR "ischemic stroke"[All Fields] OR "Brain Infarction"[MeSH Terms] OR "brain infarction*"[All Fields] OR "infarction* brain"[All Fields] OR "brain infarct*"[All Fields] OR "infarct* brain"[All Fields] OR "venous infarction brain"[All Fields] OR "brain venous infarction*"[All Fields] OR "venous infarctions brain"[All Fields] OR "venous brain infarction*"[All Fields] OR "Anterior Cerebral Circulation Infarction"[All Fields] OR "Anterior Circulation Brain Infarction"[All Fields] OR "brain infarction posterior circulation"[All Fields] OR "Posterior Circulation Brain Infarction"[All Fields] OR "Embolic Stroke"[MeSH Terms] OR "Embolic Strokes"[All Fields] OR "stroke* embolic"[All Fields] OR "cardioembolic stroke*"[All Fields] OR "stroke* cardioembolic"[All Fields] OR "cardio embolic stroke*"[All Fields] OR "Cardio embolic Stroke"[All Fields] OR "stroke* cardio embolic"[All Fields])
Virtual Health Library (VHL)<br>**848** (excluding MEDLINE)	((mh:"Doença de Chagas" OR ("Doença de Chagas") OR ("Chagas Disease") OR ("Enfermedad de Chagas") OR ("Infecção por Trypanosoma cruzi") OR ("Mal de Chagas") OR ("Tripanossomose Sul-Americana") OR ("Tripanossomíase Americana") OR ("Tripanossomíase Sul-Americana") OR mh:c01.610.752.300.900.200 OR mh:c01.920.625 OR mh:sp4.012.148.149*) AND (mh:"Acidente Vascular Cerebral" OR (stroke) OR ("Accidente Cerebrovascular") OR (avc) OR ("AVC Agudo") OR (ave) OR ("Acidente Cerebral Vascular") OR ("Acidente Cerebrovascular") OR ("Acidente Vascular Cerebral (AVC)") OR ("Acidente Vascular Cerebral Agudo") OR ("Acidente Vascular Encefálico") OR ("Acidente Vascular do Cérebro") OR ("Acidentes Cerebrais Vasculares") OR ("Acidentes

	Cerebrovasculares") OR ("Acidentes Vasculares Cerebrais") OR (apoplexia) OR ("Apoplexia Cerebral") ("Apoplexia Cerebrovascular") OR ("Icto Cerebral") OR ("Ictus Cerebral") OR ("ischemic stroke") OR mh: c10.228.140.300.775 OR mh: c14.907.253.855*)) OR (mh: "Infarto Encefálico" OR ("Infarto Encefálico") OR ("Brain Infarction") OR ("Infarto do Encéfalo") OR ("Infarto Venoso do Encéfalo") OR ("Infarto Venoso Cerebral") OR ("Infarto Venoso Encefálico") OR mh:c10.228.140.300.150.477 OR mh:c10.228.140.300.775.200 OR mh:c14.907.253.092.477 OR mh:c14.907.253.855.200 OR mh:c23.550.513.355.250 OR mh:c23.550.717.489.250*) OR (mh:"AVC Embólico" OR ("Accidente Cerebrovascular Embólico") OR ("Embolic Stroke") OR ("Acidentes Vasculares Cardioembólicos") OR ("Acidente Vascular Cardioembólico") OR mh: c10.228.140.300.775.400.375 OR mh:c14.907.253.855.400.375*) AND db:("LILACS" OR "CUMED" OR "BINACIS" OR "LIPECS" OR "SES-SP" OR "colecionaSUS" OR "BDENF" OR "VETINDEX" OR "MedCarib" OR "BBO" OR "BDNPAR" OR "BRISA" OR "INDEXPSI" OR "MINSALCHILE") AND instance:"lilacsplus"
Web of Science<br>**223**	(TS=("Chagas Disease" OR "American Trypanosomiasis" OR "Trypanosomiasis, America" OR "Trypanosomiasis, South American" OR "South American Trypanosomiasis" OR "Trypanosoma cruzi Infection" OR "Infection*, Trypanosoma cruzi" OR "Trypanosoma cruzi Infections" OR "Chagas' Disease") AND (TS=("Stroke" OR "Strokes" OR "Cerebrovascular Accident*" "CVA* Cerebrovascular Accident" OR "Cerebrovascular Apoplexy" OR "Apoplexy, Cerebrovascular" OR "Vascular Accident, Brain" OR "Brain Vascular Accident*" OR "Vascular Accidents, Brain" OR "Cerebrovascular Stroke*" OR "Stroke*, Cerebrovascular" OR Apoplexy OR "Cerebral Stroke*" OR "Stroke*, Cerebral" OR "Stroke*, Acute" OR "Acute Stroke*" OR "Cerebrovascular Accident*, Acute" OR "Acute Cerebrovascular Accident*" OR "ischemic stroke") OR TS=("Brain Infarction" OR "brain infarction* OR "infarction* brain" OR "brain infarct*" OR "infarct* brain" OR "venous infarction brain" OR "brain venous infarction*" OR "venous infarctions brain" OR "venous brain infarction*" OR "Anterior Cerebral Circulation Infarction" OR "Anterior Circulation Brain Infarction" OR "brain infarction posterior circulation"OR "Posterior Circulation Brain Infarction" OR "Embolic Stroke" OR "Embolic Strokes" OR "stroke* embolic" OR "cardioembolic stroke*" OR "stroke* cardioembolic" OR "cardio embolic stroke*" OR "Cardio embolic Stroke OR "stroke* cardio embolic"))
Embase<br>**233**	('chagas disease':ti,ab,kw OR 'american trypanosomiasis':ti,ab,kw OR 'trypanosomiasis, america':ti,ab,kw OR 'trypanosomiasis, south american':ti,ab,kw OR 'south american trypanosomiasis':ti,ab,kw OR 'trypanosoma cruzi infection':ti,ab,kw OR 'infection*, trypanosoma cruzi':ti,ab,kw OR 'trypanosoma cruzi infections':ti,ab,kw) AND ('stroke':ti,ab,kw OR 'strokes':ti,ab,kw OR 'cerebrovascular accident*':ti,ab,kw OR 'cva* cerebrovascular accident':ti,ab,kw OR 'cerebrovascular apoplexy':ti,ab,kw OR 'apoplexy, cerebrovascular':ti,ab,kw

	OR 'vascular accident, brain':ti,ab,kw OR 'brain vascular accident*':ti,ab,kw OR 'vascular accidents, brain':ti,ab,kw OR 'cerebrovascular stroke*':ti,ab,kw OR 'stroke*, cerebrovascular':ti,ab,kw OR 'apoplexy':ti,ab,kw OR 'cerebral stroke*':ti,ab,kw OR 'stroke*, cerebral':ti,ab,kw OR 'stroke*, acute':ti,ab,kw OR 'acute stroke*':ti,ab,kw OR 'cerebrovascular accident*, acute':ti,ab,kw OR 'acute cerebrovascular accident*':ti,ab,kw OR 'ischemic stroke':ti,ab,kw OR 'brain infarction':ti,ab,kw OR 'brain infarction*':ti,ab,kw OR 'infarction* brain':ti,ab,kw OR 'brain infarct*':ti,ab,kw OR 'infarct* brain':ti,ab,kw OR 'venous infarction brain':ti,ab,kw OR 'brain venous infarction*':ti,ab,kw OR 'venous infarctions brain':ti,ab,kw OR 'venous brain infarction*':ti,ab,kw OR 'anterior cerebral circulation infarction':ti,ab,kw OR 'anterior circulation brain infarction':ti,ab,kw OR 'brain infarction posterior circulation':ti,ab,kw OR 'posterior circulation brain infarction':ti,ab,kw OR 'embolic stroke':ti,ab,kw OR 'embolic strokes':ti,ab,kw OR 'stroke* embolic':ti,ab,kw OR 'cardioembolic stroke*' OR 'stroke* cardioembolic' OR 'cardio embolic stroke*':ti,ab,kw OR 'cardio embolic stroke':ti,ab,kw OR 'stroke* cardio embolic':ti,ab,kw) AND [embase]/lim
CAPES Catalog of Theses and Dissertations<br>**29**	(Chagas Disease) AND (Stroke OR Brain Infarction OR cardioembolic stroke)

Supplement 2 – Study Selection and Excluded Studies

Justifications for the exclusion of studies that were submitted for full-text reading.

Articles identified through database searches:

Reference	Decision	Reason for Exclusion	justification for exclusion
1. FJ CA, AP V, M M, TA H. American trypanosomiasis (Chagas' disease): an unrecognised cause of stroke. Journal of neurology, neurosurgery, and psychiatry. abril de 2003;74(4):516–8.	deleted	Absence of a comparison group	Inadequate population (all participants had stroke)
2. Tinone G. Analysis of the frequency of embolic cerebrovascular stroke in patients with Chagas disease. ARQUIVOS DE NEUROPSIQUIATRIA. junho de 1997;55(2):341–2.	deleted	Absence of a comparison group	Inadequate population (all participants had Chagas disease, without a comparison group)
3. JMC M, DL SM, BCG S, PAP J, J OF. Anticoagulation in patients with cardiac manifestations of Chagas disease and cardioembolic ischemic stroke. Arquivos de neuro-psiquiatria. janeiro de 2018;76(1):22–5	deleted	Absence of a comparison group	Inadequate population (The study focuses only on patients who already have Chagas disease and stroke to evaluate anticoagulant use; it does not compare CD vs non-CD)
4. Montanaro VVA, Hora TF, Guerra AA, Silva GS, Bezerra RP, Oliveira-Filho J, Santos LSB, de Melo ES, Alves de Andrade LP, Junior WAO, de Meira FCA, Nunes MDCP, Oliveira EMJ, de Freitas GR. Artificial Inteligence-Based Decision for the Prediction of Cardioembolism in Patients with Chagas Disease and Ischemic Stroke. J Stroke Cerebrovasc Dis. 2021 Oct;30(10):106034. doi: 10.1016/j.jstrokecerebrovasdis.2021.106034. Epub 2021 Aug 13. PMID: 34399284.	deleted	Absence of a comparison group	Inadequate population (Development of a predictive model in patients who already have Chagas disease and ischemic stroke)
5. JO DJ, MO da CR, AC de S, LJ K, LA de SD, TC T, et al. Assessment of the source of ischemic	deleted	Absence of a	Inadequate population

cerebrovascular events in patients with Chagas disease. International journal of cardiology. outubro de 2014;176(3):1352–4.		comparison group	(Assesses sources of embolism only in patients with Chagas disease)
6. Lopes ER, Marquez JO, Costa Neto B da, Menezes AAC, Chapadeiro E. Associação entre acidentes vasculares encefálicos e doença de Chagas. Rev Soc Bras Med Trop. junho de 1991;24(2):101–4.	deleted	Absence of a comparison group	Inadequate population (autopsy study)
7. HT M, GJ V, GM M, MF BS, A PF, JA MN, et al. Association of left ventricular abnormalities with incident cerebrovascular events and sources of thromboembolism in patients with chronic Chagas cardiomyopathy. Journal of cardiovascular magnetic resonance : official journal of the Society for Cardiovascular Magnetic Resonance. novembro de 2022;24(1):52.	deleted	Absence of a comparison group	Inadequate population (Assesses incident events only within a cohort of patients with Chagas cardiomyopathy, without a non-CD comparator arm)
8. Freitas E, Sampaio E, Oliveira M, Oliveira L, Guimaraes M, Pinheiro J, et al. Atrial High-Rate Episodes and Their Association with Cerebral Ischemic Events in Chagasic Patients. ARQUIVOS BRASILEIROS DE CARDIOLOGIA. dezembro de 2020;115(6):1072–9.	deleted	Absence of a comparison group	Inadequate population (Focused exclusively on patients with Chagas disease and cardiac implantable electronic devices)
9. Carod-Artal F, Silveira L, Pedro A, Brasilia V, Kummer W, Mesquita H. Awareness of stroke risk in Chagas disease' stroke patients. NEUROLOGY. março de 2007;68(12):A360–A360.	deleted	Absence of a comparison group	Inadequate population (Although it compares patients with and without CD, all included participants had already experienced stroke. The objective is to compare risk awareness and clinical profile between stroke-CD vs stroke–non-CD, which does not allow estimation of stroke risk in the general CD

			population)
10. FJ CA, S RL, AP V. Awareness of stroke risk in chagasic stroke patients. Journal of the neurological sciences. dezembro de 2007;263(1):35–9.	deleted	Absence of a comparison group	Inadequate population (Although it compares patients with and without CD, all included participants had already experienced stroke. The objective is to compare risk awareness and clinical profile between stroke-CD vs stroke–non-CD, which does not allow estimation of stroke risk in the general CD population)
11. Ribeiro A, Matos D, Costa M. B-type Natriuretic Predicts 10-year Stroke Mortality in Community-Dwelling Elderly with Chagas Disease (the Bambuii Study). CIRCULATION. novembro de 2010;122(21)	deleted	Subanalysis	Subanalysis of reference 25
12. LE E, AY SG, LZ R, F SS, M N, L A, et al. Beyond cardiac embolism and cryptogenic stroke: unveiling the mechanisms of cerebrovascular events in Chagas disease. Lancet regional health Americas. outubro de 2025;50:101203	deleted	Ineligible study design	Ineligible study type (Review article/perspective on pathophysiological mechanisms)
13. VG M, L R, ACB de L, RR F, LS O, LM N, et al. Biomarkers and Echocardiographic Predictors of Cardiovascular Outcome in Patients With Chronic Chagas Disease. Journal of the American Heart Association. junho de 2023;12(12):e028810	deleted	Absence of a comparison group	Inadequate population (Study of outcome predictors only in patients with chronic Chagas disease)
14. Moncayo J. Causas coexistentes de infarto cerebral. Reflexiones (Impresa). maio de 2002;7(1):29–37	deleted	Ineligible	Ineligible study type (Reflective

		study design	article/descriptive case series)
15. Oliveira-Filho J, Neville IS, Cincura C, Menezes DF, Ribeiro-dos-Santos Jr. V, Pimentel LS, et al. Cerebral embolism and death are associated with chagas disease cardiopathy: A transcranial doppler studywith long-term outcome assessment. Cerebrovasc Dis. 2010;29:16.	deleted	Absence of a comparison group	Inadequate population (Assesses microembolism by transcranial Doppler only in patients with Chagas cardiomyopathy)
16. Montanaro VVA, Hora TF, da Silva CM, de Viana Santos CV, Lima MIR, de Jesus Oliveira EM, de Freitas GR. Cerebral infarct topography of atrial fibrillation and Chagas disease. J Neurol Sci. 2019 May 15;400:10-14. doi: 10.1016/j.jns.2019.03.002. Epub 2019 Mar 12. PMID: 30878634.	included		
17. R A, JA da M, G M, I G, A M. Cerebral infarction in autopsies of chagasic patients with heart failure. Arquivos brasileiros de cardiologia. outubro de 2003;81(4):414–6, 411–3.	deleted	Absence of a comparison group	Inadequate population (Analyzes only a cohort of patients with Chagas disease and heart failure, without a non-Chagas comparator group)
18. SC DC, PC M, MB S, S BJ. [Cerebrovascular disorders in Uberlandia: II. Epidemiology and clinic medicine]. Arquivos de neuro-psiquiatria. junho de 1986;44(2):139–46	deleted	Absence of a comparison group	Insufficient data for 2×2 extraction (Local descriptive epidemiological study that does not provide the total denominator of patients without the disease to construct the contingency table)
19. del Brutto O. Cerebrovascular disease in the tropics. REVISTA DE NEUROLOGIA. outubro de 2001;33(8):750–62.	deleted	Ineligible study design	Ineligible study type (Review article)

20. Meira F, Teixeira A, Do Carmo Nunes M. Chagas disease and stroke: Frequency and clinical correlates. Neurology [Internet]. 2016;86(16). Disponível em: https://www.embase.com/search/results?subaction=viewrecord&id=L72250515&from=export U2 - L72250515	deleted	Absence of a comparison group	The analysis started from stroke; the study did not present comparison groups
21. Viana L, Reis F, Lacerda A, Vieira-de-Melo R, Jesus P, Dias J, et al. Chagas disease cohort study: Long-term predictors of stroke and death. STROKE. fevereiro de 2006;37(2):672–672.	deleted	Absence of a comparison group	Inadequate population (Focused on long-term predictors within a cohort exclusively of patients with Chagas disease)
22. Cerqueira-Silva T, Gonçalves BM, Pereira CB, Porto LM, Marques ME, Santos LS, Oliveira MA, Félix IF, de Sousa PRP, Muiños PJ, Maia RM, Catto MB, Andrade AL, Jesus PA, Aras R, Oliveira-Filho J. Chagas disease is an independent predictor of stroke and death in a cohort of heart failure patients. Int J Stroke. 2022 Feb;17(2):180-188. doi: 10.1177/17474930211006284. Epub 2021 Apr 7. PMID: 33724086.	included		
23. Gonc¸alves BM, Oliveira-Filho J, Andrade AL, Barreto-Neto N, Ferreira IL, Fernandes RD, et al. Chagas disease is an independent risk factor for stroke or death: Long-term results from a hospital-based cohort. Stroke [Internet]. 2016;47. Disponível em: https://www.ovid.com/journals/stro/abstract/00007670-201602001-00869~abstract-wp219-chagas-disease-is-an-independent-risk-factor?redirectionsource=fulltextview	deleted	Insufficient data	Full text requested from the author; no response was received; therefore, the study was excluded because it was a conference abstract and did not provide all information required for inclusion
24. J OF, LC V, RM V de M, F F, JA T, FA V, et al. Chagas disease is an independent risk factor for stroke: baseline characteristics of a Chagas Disease cohort. Stroke. setembro de 2005;36(9):2015–7	included		

25. Lima-Costa MF, Matos DL, Ribeiro AL. Chagas disease predicts 10-year stroke mortality in community-dwelling elderly: the Bambui cohort study of aging. Stroke. 2010 Nov;41(11):2477-82. doi: 10.1161/STROKEAHA.110.588061. Epub 2010 Sep 23. PMID: 20864663.	included		
26. Cougo-Pinto PT, Dos Santos BL, Dias FA, Camilo MR, Alessio-Alves FF, Barreira CM, et al. Chagas disease related stroke: Safety of intravenous thrombolysis and endovascular treatment. Stroke [Internet]. 2014;45. Disponível em: https://www.embase.com/search/results? subaction=viewrecord&id=L71465334&from=export U2 - L71465334	deleted	Absence of a comparison group	Inadequate population (Study focused on the safety of acute-phase treatments in patients who had already experienced stroke)
27. ES de M, R de PB, AC de H, MJ LES, PP T, TFL da S, et al. Chagas disease stroke and associated risk factors: A case-control study. Journal of stroke and cerebrovascular diseases : the official journal of National Stroke Association. janeiro de 2024;33(1):107463.	deleted	Absence of a comparison group	The analysis started from stroke; the study did not present comparison groups
28. J VG, C M, C SS, M VG, E OP. Chagas disease as an underrecognized cause of stroke: implications for public health. Frontiers in medicine. 2024;11:1473425.	deleted	Ineligible study design	Ineligible study type (Perspective/narrative review)
29. S Y, V W. Chagas disease, cardioembolic ischemic stroke, INR control and bleeding. Arquivos de neuro-psiquiatria. janeiro de 2019;77(1):65.	deleted	Absence of a comparison group	Inadequate population (Focus on INR control and bleeding in patients with Chagas disease and stroke; no non-CD control group)
30. LC P, AL R, RA V, AL T. Chagas disease: independent risk factor for stroke. Stroke. dezembro de 2009;40(12):3691–4	deleted	Absence of a comparison group	The analysis started from stroke; the study did not present comparison groups
31. FJ CA, AP V, TA H, LG N. Chagasic cardiomyopathy is independently associated with ischemic stroke in	deleted	Absence of a comparison	Inadequate population (The study analyzes stroke

Chagas disease. Stroke. maio de 2005;36(5):965–70.		group	risk factors only within a cohort of patients who already have Chagas disease, comparing those with and without cardiomyopathy; it lacks a non-Chagas control group)
32. Carod-Artal FJ, Ferreira-Coral L, Stieven Trizotto D, Menezes-Moreira C. Chagasic ischemic stroke is not associated with worse prognosis in disability and quality of life. Cerebrovasc Dis. 2010;29:271.	deleted	Absence of a comparison group	Inadequate population (Focused on prognosis and quality of life in patients who had already experienced stroke and have Chagas disease; it does not compare incidence between CD vs non-CD)
33. Spina Franca A, Yasuda N. Chronic Chagas cardiopathy and cerebrovascular insults. INCIDENCIA DE ACIDENTE CEREBRO VASCULAR EMBOLICO NA CARDIOPATIA CHAGASICA CRONICA. 1974;32(3):195–8.	deleted	Absence of a comparison group	Inadequate population (Case series of patients with chronic Chagas cardiomyopathy to assess stroke frequency, without a disease-free comparator group)
34. RB B, R DJ, LB C. Comments on Etiological Classification of Stroke in Patients with Chagas Disease Using TOAST, Causative Classification System TOAST, and ASCOD Phenotyping. Journal of stroke and cerebrovascular diseases : the official journal of National Stroke Association. maio de 2018;27(5):1434.	deleted	Ineligible study design	Ineligible study type (Commentary/editorial on stroke classification systems)
35. Oliveira LMB, Ornellas ACP, Neville IS, Cincura C, Menezes DF, Jesus PAP, et al. Cyclooxygenase 2 (COX-2) but not arachidonate 5- lipoxygenase-activating protein (ALOX5AP) gene polymorphism is associated with	deleted	Absence of a comparison	Inadequate population (Genetic polymorphism study in a specific

ischemic stroke in a population with cardiomyopathy and Chagas disease. Cerebrovasc Dis. 2009;27:121–2		group	population of patients with Chagas cardiomyopathy, focused on genetic markers rather than risk comparison between CD and non-CD for meta-analysis)
36. Oliveira-Filho J, Ornellas ACP, Ribeiro-dos-Santos Jr. V, Pimentel LS, Silva PMFS, Neville IS, et al. Cycloxygenase-2 gene polymorphism, but not 5-lipoxygenase activating protein polymorphism, is associatedwith ischemic stroke and chagas disease. Cerebrovasc Dis. 2010;29:176.	deleted	Absence of a comparison group	Inadequate population (Genetic polymorphism study in a specific population of patients with Chagas cardiomyopathy, focused on genetic markers rather than risk comparison between CD and non-CD for meta-analysis)
37. AC S, MO R, AL T, JO DJ, LA S, MC N. Depressive symptoms and disability in chagasic stroke patients: impact on functionality and quality of life. Journal of the neurological sciences. janeiro de 2013;324(1):34–7.	deleted	Absence of a comparison group	Inadequate population (Assesses depressive symptoms and functionality only in patients who already had stroke and have Chagas disease)
38. FSNS M, MFF M, RS S, SS X, PEAA do B, RM S, et al. Discussing the Score of Cardioembolic Ischemic Stroke in Chagas Disease. Tropical medicine and infectious disease [Internet]. maio de 2020;5(2). Disponível em: https://pubmed.ncbi.nlm.nih.gov/32466425/	deleted	Absence of a comparison group	Inadequate population (Technical discussion on the application of risk scores specifically for the Chagas population)
39. Rey RC, Lepera SM, Kohler G, Monteverde DA, Sica RE. Embolia cerebral de origen cardíaco. Medicina	deleted	Absence of a comparison	Inadequate population (Analyzes cardioembolic

(BAires). 1992;52(3):202–6		group	sources in patients with stroke, but does not provide comparative 2×2 data between those exposed and not exposed to CD in a base or control population)
40. Mina Morales F, Castro Flores JA. Enfermedad de Chagas y embolia cerebral. Arch boliv med. 1995;2(47):2–6.	deleted	Ineligible study design	Ineligible study type (Descriptive case report/series)
41. Auger S, Storino R, Iglesias Ordoñez O, Urrutia MI, Sanmartino M, Romero D, et al. Emergências em pacientes com doença de Chagas na cidade de Buenos Aires, Argentina. Rev Soc Bras Med Trop. dezembro de 2002;35(6):609–16.	deleted	Absence of a comparison group	Inadequate population (Study focused on the emergency care profile of patients with Chagas disease; the primary outcome is not comparison of stroke incidence with non-Chagas patients)
42. REY R, LEPERA S, KOHLER G, MONTEVERDE D, SICA R. EMBOLIC STROKES OF CARDIAC ORIGIN. MEDICINA-BUENOS AIRES. 1992;52(3):202–6.	deleted	Absence of a comparison group	Inadequate population (Analyzes cardioembolic sources in patients with stroke, but does not provide comparative 2×2 data between those exposed and not exposed to CD in a base or control population)
43. VVA M, TF H, CM da S, CV de VS, MIR L, EM de JO, et al. Epidemiology of concurrent Chagas disease and ischemic stroke in a population attending a multicenter quaternary rehabilitation network in Brazil. Neurological sciences : official journal of the Italian Neurological Society and of the Italian Society of Clinical	deleted	Absence of a comparison group	Inadequate population (Cross-sectional study in a rehabilitation network

Neurophysiology. dezembro de 2019;40(12):2595–601			describing the prevalence of Chagas disease among individuals who already had stroke, without a non-stroke control group to determine population-level relative risk or odds ratio as per protocol)
44. Sousa AS de, Xavier SS, Freitas GR de, Hasslocher-Moreno A. Estratégias de prevenção do acidente vascular encefálico cardioembólico na doença de Chagas. Arq bras cardiol. novembro de 2008;91(5):306–10.	deleted	Ineligible study design	Ineligible study type (Review article/guidelines on prevention strategies)
45. R de PB, MA de MA, AB C, DLG R, GS S. Etiological Classification of Stroke in Patients with Chagas Disease Using TOAST, Causative Classification System TOAST, and ASCOD Phenotyping. Journal of stroke and cerebrovascular diseases : the official journal of National Stroke Association. dezembro de 2017;26(12):2864–9.	deleted	Absence of a comparison group	Inadequate population (The study focuses on etiological classification of patients who simultaneously have Chagas disease and stroke, precluding comparison of incidence/risk between CD and non-CD groups)
46. Mina Morales F, Castro Flores JA. Enfermedad de Chagas y embolia cerebral. Arch boliv med. 1995;2(47):2–6.	deleted	Ineligible study design	Ineligible study type (Descriptive case report or case series; reference identical to 40)
47. VVA M, GS S, J OF, OM PN, M do CPN, RP B, et al. Exploring the Chagas Disease-Stroke "Connection": Findings from a Large Multicenter Study. Cerebrovascular diseases (Basel, Switzerland). 2024;53(6):729–33.	deleted	Absence of a comparison group	All participants had Chagas disease and stroke
48. Fernandes HA, Amaral DAS, Nascimento EGC do, Martins RR, Souza AS de, Andrade C de M. Forma clínica indeterminada da doença de Chagas e o risco potencial de evento isquêmico. Rev baiana saúde pública.	deleted	Absence of a comparison	Inadequate population (Assesses risk markers only in the indeterminate

janeiro de 2021;45(1):153–65.		group	form of the disease, without a non-Chagas control group for comparison of clinical stroke outcomes)
49. FJ CA. [Globalization, stroke and Chagas disease on the hundredth anniversary of its discovery]. Neurologia (Barcelona, Spain). julho de 2009;24(6):431–2.	deleted	Ineligible study design	Ineligible study type (Editorial/commentary)
50. ACJ S, ND Q, JEFA S, MDCP N, JA P, EC S, et al. Hospitalizations due to stroke and their relationship with Chagas disease: a population-based analytical study using data from the Hospital Information System of the Brazilian National Health System, Minas Gerais, Brazil, 2022. Epidemiologia e servicos de saude : revista do Sistema Unico de Saude do Brasil. 2025;35:e20240901.	deleted	Absence of a comparison group	Insufficient data for 2×2 extraction (Ecological study based on SIH/SUS that quantifies stroke hospitalizations associated with the Chagas code, but does not provide the total denominator of the population at risk without the disease to calculate incidence)
51. Souza A, Júnior JOD, Teixeira AL, Da Costa Rocha MO, Sousa LAP, Do Carmo Pereira Nunes M. Impact of ischemic cerebrovascular events on quality of life and functional performance in patients with chagas disease. Trop Med Int Health. 2011;16:317–8.	deleted	Absence of a comparison group	Inadequate population (Focused on functional impact and quality of life in patients who already have Chagas disease and stroke)
52. A SF, N Y. [Incidence of embolic cerebrovascular accidents in chronic Chagas' cardiopathies]. Arquivos de neuro-psiquiatria. setembro de 1974;32(3):195–8.	deleted	Absence of a comparison group	Inadequate population (Reference identical to 33; case series without a non-CD control group)
53. Pinazo M, Tassies D, Muñoz J, Fisa R, Posada E, Monteagudo J, et al. Hypercoagulability biomarkers in Trypanosoma cruzi-infected patients. THROMBOSIS AND HAEMOSTASIS. outubro de 2011;106(4):617–23.	deleted	Inappropriate outcome	Inadequate outcome (The study evaluates

			biomarkers of hypercoagulability rather than the clinical occurrence of stroke)
54. NX P, MA TH, E M, FE LS. Immune response, nitric oxide, autonomic dysfunction and stroke: a puzzling linkage on Trypanosoma cruzi infection. Medical hypotheses. maio de 2002;58(5):374–7.	excl uid	Ineligible study design	Ineligible study type (Medical hypothesis/theoretical article)
55. PM G, CM de A, DF N, N de SP, TB Q, GL MC, et al. Inflammation Enhances the Risks of Stroke and Death in Chronic Chagas Disease Patients. PLoS neglected tropical diseases. abril de 2016;10(4):e0004669	deleted	Absence of a comparison group	Inadequate population (Studies the association between inflammation and stroke within a cohort exclusively of patients with Chagas disease, without a non-Chagas comparator group)
56. Mutarelli A, Lage TR, Tupinambás J, De Padua LB, Ferreira A, Da Silva JLP, et al. INVESTIGATING CLINICAL FEATURES AND PREDICTORS OF ISCHEMIC STROKE IN A LARGE COHORT OF PATIENTS WITH CHAGAS CARDIOMYOPATHY: EXPANDING THE FOCUS BEYOND CARDIOMYOPATHY SEVERITY. J Am Coll Cardiol. 2024;83(13):2314.	deleted	Absence of a comparison group	Inadequate population (Cohort focused on stroke predictors in patients with Chagas cardiomyopathy; absence of a non-CD arm)
57. PV W, RM F, Jr EJ, AC DS, Jr MW, AA B. Involvement of the central nervous system in the chronic form of Chagas' disease. Journal of the neurological sciences. junho de 2008;269(1):152–7	deleted	Absence of a comparison group	Inadequate population (Descriptive study of CNS involvement by MRI only in patients with chronic Chagas disease)
58. Jr TA, MM T. Ischaemic stroke in chagasic patients. Journal of neurology, neurosurgery, and psychiatry. março de 2004;75(3):510; author reply 510	deleted	Ineligible study design	Ineligible study type (Letter to the editor/authors' response)

59. JE P. Ischemic cerebral changes in the chronic chagasic cardiopathy. Arquivos de neuro-psiquiatria. junho de 1984;42(2):105–15.	deleted	Absence of a comparison group	Inadequate population (Study focused only on ischemic brain changes in patients who already have Chagas cardiomyopathy, without a non-Chagas comparator arm)
60. Do Carmo P Nunes M, Colosimo EA, Almeida FR, SantosJunior PC, Martins MAP, De Paula TMN, et al. Ischemic cerebrovascular events are independent predictors of death in patients with chagas disease: Time varying covariate analysis. Circulation [Internet]. 2017;136. Disponível em: https://www.embase.com/search/results?subaction=viewrecord&id=L619984251&from=export U2 - L619984251	deleted	Absence of a comparison group	Inadequate population (Studies that assess stroke as a predictor of death or classify stroke type only in patients who already have Chagas disease)
61. MC N, MM B, AL R, FB B, MO R. Ischemic cerebrovascular events in patients with Chagas cardiomyopathy: a prospective follow-up study. Journal of the neurological sciences. março de 2009;278(1):96–101.	deleted	Absence of a comparison group	Inadequate population (Studies that assess stroke as a predictor of death or classify stroke type only in patients who already have Chagas disease)
62. VV M, CM da S, CV de VS, MI L, EM N, GR de F. Ischemic stroke classification and risk of embolism in patients with Chagas disease. Journal of neurology. dezembro de 2016;263(12):2411–5.	deleted	Absence of a comparison group	Inadequate population (Studies that assess stroke as a predictor of death or classify stroke type only in patients who already have Chagas disease)
63. Tinone G, Yamamoto F, Comerlatti L, Massaro A, Ianni B, SpinaFranca A, et al. Ischemic stroke in Chagas' disease. NEUROLOGY. fevereiro de 1996;46(2):3045–3045	deleted	Absence of a comparison group	Insufficient data for 2×2 extraction (Conference abstract that does not provide the population denominator of control

			groups for risk calculation)
64. Pedrosa RC. Mecanismo causante de Acidente Vascular Cerebral Embólico na Cardiopatia Chagásica Crônica: Disfunção Autonômica, uma Hipótese de Trabalho. Arq bras cardiol. dezembro de 2020;115(6):1080–1.	deleted	Ineligible study design	Ineligible study type (Hypothesis article/editorial commentary)
65. VVA M, TF H, CM da S, CVV S, MIR L, EM N, et al. Mortality and stroke recurrence in a rehabilitation cohort of patients with cerebral infarcts and Chagas disease. Eur Neurol. 2018;79(3):177–84.	deleted	Absence of a comparison group	Inadequate population (Study conducted in a rehabilitation cohort in which all patients had already experienced stroke; focuses on recurrence rather than primary comparative incidence between CD and non-CD)
66. Martins-Melo FR, Ramos AN Jr, Alencar CH, Heukelbach J. Multiple causes of death related to Chagas' disease in Brazil, 1999 to 2007. Rev Soc Bras Med Trop. 2012 Sep;45(5):591–6.	deleted	Absence of a comparison group	Inadequate population (Epidemiological study of causes of death based on SIM/DATASUS; does not provide individual-level 2×2 incidence data for stroke among exposed and unexposed)
67. Oliveira-Filho J, Muinos PJR, Pereira CB, Porto LM, Gonçalves BMM, Andrade AL, et al. Neuronal depopulation, not silent cerebrovascular disease, mediate cognitive dysfunction in Chagas disease. Eur Stroke J. 2018;3(1):480.	deleted	Inappropriate outcome	Inadequate outcome (The primary focus is cognitive dysfunction rather than clinical stroke incidence for risk meta-analysis)

68. EES S, MMC O, R A. Occurrence of Stroke and Reduced Ejection Fraction in Patients with Chagas Disease. Arquivos brasileiros de cardiologia. março de 2018;110(3):297	deleted	Ineligible study design	Ineligible study type (Letter to the editor/commentary)
69. Nunes MC, Barbosa MM, Rocha MO. Peculiar aspects of cardiogenic embolism in patients with Chagas' cardiomyopathy: a transthoracic and transesophageal echocardiographic study. *J Am Soc Echocardiogr*. 2005;18(7):761–7.	deleted	Absence of a comparison group	Inadequate population (Assesses echocardiographic aspects only in patients with Chagas disease)
70. Cardoso RC, Silva CVC, Barbosa VLPP, de Sousa PRSP, Nunes MCP, Ferreira KL, et al. Pilot randomized controlled trial of acetylsalicylic acid to reduce cerebral microembolism in Chagas heart failure. *Arq Neuropsiquiatr*. 2025;83(10):1–7.	deleted	Ineligible study design	Ineligible study type (Pilot clinical trial on aspirin use specifically in patients with Chagas heart failure)
71. Carod-Artal FJ. Policy implications of the changing epidemiology of Chagas disease and stroke. *Stroke*. 2013;44(8):2356–60.	deleted	Ineligible study design	Ineligible study type (Review and opinion article on public policy)
72. Silva CP, Del Carlo CH, Oliveira Junior MT, Scipioni A, Strunz-Cassaro C, Ramirez JAF, et al. Por que os portadores de cardiomiopatia chagásica têm pior evolução que os não-chagásicos? *Arq Bras Cardiol*. 2008;91(6):389–94.	deleted	Inappropriate outcome	The study did not evaluate stroke as an outcome
73. Cardoso S, Guedes NJ. Platelets, Macrophages, and Thromboinflammation in Chagas Disease. *J Inflamm Res*. 2022;15:5689–706.	deleted	Ineligible study design	Ineligible study type (Pathophysiological review article)
74. Lemos TAR, Rocha MOC, Teixeira JT, Barbosa FA, de Paula LB, et al. Predictors of ischemic stroke in Chagas disease: Insights into mechanisms beyond cardiomyopathy severity. *Int J Cardiol*. 2025;418:132628.	deleted	Absence of a comparison group	Inadequate population (The study focuses on stroke predictors exclusively within a cohort of patients with Chagas disease, without a non-Chagas comparator arm)

75. Nunes MC, Kreuser LJ, Ribeiro AL, Sousa GR, Costa HS, Barbosa FA, et al. Prevalence and risk factors of embolic cerebrovascular events associated with Chagas heart disease. *Glob Heart*. 2015;10(3):151–7	deleted	Absence of a comparison group	Inadequate population (Analyzes prevalence and risks only among patients with Chagas cardiomyopathy)
76. Lima Freitas E, Sampaio ES, Oliveira MMC, Albuquerque GSB, Guimarães MSS, Pinheiro JO, et al. Prevalence of ischemic stroke in patients with Chagas disease using cardiac implantable electronic devices. *Eur Heart J*. 2017;38:1147–8.	deleted	Absence of a comparison group	Inadequate population (Specific subgroup of patients with Chagas disease and electronic devices, without a non-CD control group for incidence comparison)
77. Carod-Artal F. Prevalence of seizures following ischemic stroke in patients with Chagas disease. *Neurology*. 2012;78(1).	deleted	Absence of a comparison group	Inadequate population (Focus on epileptic seizures in patients who already have Chagas disease and have experienced stroke)
78. Sousa AS, Xavier SS, Figueiredo GR, Horta HM. Prevention strategies of cardioembolic ischemic stroke in Chagas' disease. *Arq Bras Cardiol*. 2008;91(5):306–10.	deleted	Ineligible study design	Ineligible study type (Review article/guidelines; duplicate of reference 45)
79. Oliveira-Filho J, Reis CC, Fernandes RD, Jesus AA, Fonseca LF, Muller MC, et al. Reduced brain and cerebellar volumes correlate with cognitive deficits in Chagas disease. *Stroke*. 2014;45	deleted	Inappropriate outcome	Inadequate outcome (The study focuses on brain volumes and cognitive deficits rather than clinical stroke incidence for the 2×2 table)
80. Pinto CP, Sousa BL, Dias FA, Carvalho MR, Araújo AA, Barbosa CM, et al. Safety of IV thrombolysis in acute ischemic stroke related to Chagas disease. *Neurology*. 2013;81(20):1773–5	deleted	Absence of a comparison	Inadequate population (Focus on thrombolysis

		group	safety in patients who already had stroke; duplicate of reference 26)
81. Nogueira-Martins DM, Dias-Azevedo MF, Diniz-Azevedo VD, et al. Spirometric values associated with clinical form and risk of death and stroke in chagasic patients. *Biomed Rep*. 2022;17(5):88.	deleted	Absence of a comparison group	Inadequate population (Analyzes risks only within the population of patients with Chagas disease)
82. Oliveira-Filho J. Stroke and brain atrophy in chronic Chagas disease patients: A new theory proposition. *Dement Neuropsychol*. 2009;3(1):22–6.	deleted	Ineligible study design	Ineligible study type (Theoretical proposition/review article)
83. da Matta JA, Aras R Jr, de Macedo CR, da Cruz CG, Netto EM. Stroke correlates in chagasic and non-chagasic cardiomyopathies. PLoS One. 2012;7(4):e35116. doi: 10.1371/journal.pone.0035116. Epub 2012 Apr 16. PMID: 22523572; PMCID: PMC3327657	included		
84. Fragata J, Sampaio C. Stroke due to Chagas' cardiomyopathy or noncompaction. *Arq Bras Cardiol*. 2011;96(5):427–8.	deleted	Ineligible study design	Ineligible study type (Case report)
85. Acampa M, et al. Stroke history and Chagas disease are independent predictors of silent cerebral microembolism in patients with congestive heart failure. *Cerebrovasc Dis*. 2011;31(1):19–23.	deleted	Inappropriate outcome	Inadequate outcome (The primary outcome is silent cerebral microembolism detected by Doppler, not clinical stroke as defined by the meta-analysis criteria)
86. Barbosa JM, et al. Stroke in a chronic autochthonous chagasic patient from the Brazilian Amazon. *Rev Soc Bras Med Trop*. 2010;43(6):751–3.	deleted	Ineligible study design	Ineligible study type (Case report)
87. Bestetti RB. Stroke in a hospital-derived cohort of patients with chronic Chagas' disease. *Acta Cardiol*. 2000;55(1):33–8.	deleted	Absence of a comparison	Inadequate population (Focus on a cohort

		group	exclusively of patients with Chagas disease, without a non-CD comparator arm)
88. Carod-Artal FJ, Vargas AP, Falcón T. Stroke in asymptomatic *Trypanosoma cruzi*-infected patients. *Cerebrovasc Dis*. 2011;31(1):24–8.	deleted	Absence of a comparison group	Does not present the four absolute numbers required to calculate measures of association between CD vs non-CD for the stroke outcome (2×2 table)
89. Souza AA, Costa HS, Sousa GR, Lima MMO, Gusmão LL, Almeida F, et al. Stroke in Chagas disease: Impact of a rehabilitation program. *Eur J Prev Cardiol*. 2015;22(1 Suppl):S101.	deleted	Absence of a comparison group	Inadequate population (The study evaluates the impact of a rehabilitation program in patients who already have Chagas disease and stroke, without a non-Chagas comparator group)
90. Carod-Artal FJ, Mena M, Vargas AP. Stroke of cardioembolic origin in Chagas disease. *Rev Neurol*. 2001;33(4):311–5.	deleted	Absence of a comparison group	Inadequate population (Descriptive study of stroke causes only in patients with Chagas disease)
91. Silva F, Almeida M, Borges R, Barreto S, dos Santos B, Gonçalves L, et al. Stroke risk in two large cohorts of patients with and without Chagas disease: role of cardiac disease. *Int J Stroke*. 2025;20(2). Disponível em: https://journals.sagepub.com/doi/epub/10.1177/17474930251371440	deleted	Insufficient data	Full text requested from the author; the author reported that the work was a conference abstract and that complementary data enabling inclusion were not available

92. Carod-Artal FJ. Stroke: a neglected complication of American trypanosomiasis (Chagas' disease). *Trans R Soc Trop Med Hyg*. 2007;101(11):1075–80.	deleted	Ineligible study design	Ineligible study type (Review article)
93. M VVA. The challenge of effective secondary stroke prophylaxis in Chagas patients. *Arq Bras Cardiol*. 2024;121(5):e20240008.	deleted	Ineligible study design	Ineligible study type (Editorial/commentary on secondary prophylaxis)
94. Py M, Maciel L, Pedrosa R, Nascimento J, Medei E. THE PRESENCE OF ANTIAUTONOMIC MEMBRANE RECEPTOR ANTIBODIES DO NOT CORRELATE WITH BRAIN LESIONS IN CHAGAS' DISEASE. ARQUIVOS DE NEURO-PSIQUIATRIA. setembro de 2009;67(3):633–8	deleted	Absence of a comparison group	Inadequate population (Studies the correlation of antibodies with brain lesions only in patients with Chagas disease)
95. Busza A, Cervantes-Arslanian AM, Kase C. Thromboembolic stroke in a young woman with chagas cardiomyopathy. Neurology [Internet]. 2012;78(1). Disponível em: https://www.embase.com/search/results?subaction=viewrecord&id=L70727766&from=export U2 - L70727766	deleted	Ineligible study design	Ineligible study type (Case report)
96. FJ CA, AP V, M M, TV F da S. ["Top-of-the-basilar" syndrome and Chagas' disease]. Revista de neurologia. agosto de 2002;35(4):337–41	deleted	Ineligible study design	Ineligible study type (Case series report)
97. Carod-Artal F, da Silva T, Nunes S, Vargas A, Horan T. Thrombophilia is not a predictive factor of stroke in Chagas' disease. NEUROLOGY. abril de 2004;62(7):A240–1.	deleted	Absence of a comparison group	Inadequate population (Analyzes predictive factors of thrombophilia exclusively within the group of patients with Chagas disease)
98. Reverter JC. Thromboembolic events and prothrombotic phenomena in Chagas disease. Fenómenos tromboembólicos y estado protrombótico en la enfermedad de Chagas. 2006;8:25–7.	deleted	Ineligible study design	Ineligible study type (Review article on prothrombotic state)
99. Carod-Artal FJ. Tropical etiologies of stroke. Causas tropicales de ictus. 2008;4(2):30–8	deleted	Ineligible study design	Ineligible study type (Review article)
100. A SF, JA L, LR M, N Y. [Trypanosoma cruzi antibodies in the cerebrospinal fluid: a search	deleted	Inappropriate	Inadequate outcome (The

using complement fixation and immunofluorescence reactions]. Arquivos de neuro-psiquiatria. dezembro de 1988;46(4):374–8.		outcome	study focuses on antibody detection in cerebrospinal fluid rather than the clinical occurrence of stroke)
101. Olivo-Freites C, Sy H, Cardenas-Alvarez J, Vega-Batista F, Henao-Martínez A. Trypanosoma cruzi Central Nervous System InfectionPathogenesis, Clinical Manifestations, Diagnosis, and Treatment. CURRENT TROPICAL MEDICINE REPORTS. dezembro de 2023;10(4):186–98.	deleted	Ineligible study design	Ineligible study type (Review article)
102. FE LS, E M, M TH, N P, J P, CA S, et al. Trypanosoma cruzi-associated cerebrovascular disease: a case-control study in Eastern Colombia. Journal of the neurological sciences. janeiro de 2004;217(1):61–4.	deleted	Absence of a comparison group	The analysis started from stroke; the study did not present comparison groups
103. FJ CA. Trypanosomiasis, cardiomyopathy and the risk of ischemic stroke. Expert review of cardiovascular therapy. maio de 2010;8(5):717–28	deleted	Ineligible study design	Ineligible study type (Expert review)
104. Singh G, Njamnshi A, Sander J. Vector-borne protozoal infections of the CNS: cerebral malaria, sleeping sickness and Chagas disease. CURRENT OPINION IN NEUROLOGY. junho de 2021;34(3):439–46.	deleted	Ineligible study design	Ineligible study type (Review article)
105. Sawalha Y, Radwan S, Zaghlol R, Garcia-Garcia H. White ethnicity is associated with increased mortality and incidence of ischemic stroke in chagas disease. Circulation [Internet]. 2019;140. Disponível em: https://www.embase.com/search/results? subaction=viewrecord&id=L630920635&from=export U2 - L630920635	deleted	Absence of a comparison group	Inadequate population (Analyzes the impact of ethnicity on stroke mortality and incidence specifically within an already Chagas population; without a non-CD control group)
106. Carod Artal F. What about infection in travellers, migrants and refugees. Eur J Neurol. 2022;29:32.	deleted	Ineligible study design	Ineligible study type (Review

			article/perspective)
107. Dias JS, Lacerda AM, Vieira-de-Melo RM, Viana LC, Jesus PAP, Reis FJFB, Nitrini R, Charchat-Fichman H, Lopes AA, Oliveira-Filho J. Cognitive dysfunction in chronic Chagas disease cardiomyopathy. Dement Neuropsychol. 2009 Jan-Mar;3(1):27-33. doi: 10.1590/S1980-57642009DN30100006. PMID: 29213606; PMCID: PMC5619028.	included		
108. Jesus PA, Neville I, Cincurá C, Menezes DF, Vieira-de-Melo RM, Lacerda AM, Viana LC, Pereira DF, Ribeiro-dos-Santos V Jr, Reis FJ, Macedo C, Oliveira-Filho J. Stroke history and Chagas disease are independent predictors of silent cerebral microembolism in patients with congestive heart failure. Cerebrovasc Dis. 2011;31(1):19-23. doi: 10.1159/000319892. Epub 2010 Oct 28. PMID: 20980749.	included		

Theses and dissertations identified through database searches:

Reference	Decision	Reason for Exclusion	justification for exclusion
1. Meira FCA. Características do acidente vascular cerebral associado à doença de Chagas [dissertação]. Belo Horizonte: Universidade Federal de Minas Gerais; 2016.	delete	Absence of comparison group	all with stroke, based on the outcome.

2. Cardoso LGM. Lesões vasculares silenciosas e disfunção cognitiva em portadores de cardiomiopatia chagásica [dissertação]. Salvador: Universidade Federal da Bahia; 2021.	delete	sub-analysis	Subanalysis (this is a cross-sectional excerpt from a cohort study that has already been included in the present study (Cerqueira-Silva et al., 2021))
3. Souza, AC. Doença de Chagas e acidente vascular encefálico: impacto sobre a funcionalidade, qualidade de vida e efeitos da reabilitação [tese]: Universidade Federal de Minas Gerais; 2015.	delete	Absence of comparison group	Inadequate population, does not have the groups for comparison and the outcome is not stroke.

Articles identified in the reference list of other reviews:

Reference	Decision	Reason for Exclusion
Paixão LC, Ribeiro AL, Valácio RA, Teixeira AL. Doença de Chagas: fator de risco independente para acidente vascular cerebral. AVC. (2009) 40:3691–4. doi: 10.1161/STROKEAHA.109.560854	delete	The analysis started from the stroke/article already evaluated and located in the searches ref. 30
de Melo ES, Bezerra R, de Holanda P, de Silva AC, Travassos MJL, Silva PP, et al. Chagas disease stroke and associated risk factors: a case-control study. J Stroke Cerebrovasc Dis. 2024;33.	delete	The analysis started from the stroke/article already evaluated and located in the searches ref. 27

Articles identified in the reference list of included articles:

Reference	Decision	Reason for Exclusion
1. Aras R, da Matta JAM, Mota G, Gomes I, Melo A. Cerebral infarction in autopsies of chagasic patients with heart failure. Arq Bras Cardiol. 2003;81:414–6.	delete	1. Aras R (2003): Duplicate of Reference 17.
2. Carod-Artal FJ, Vargas AP, Falcao T. Stroke in asymptomatic Trypanosoma Cruzi-infected patients. Cerebrovasc Dis Basel Switz. 2011;31:24–8.	delete	2. Carod-Artal FJ (2011): Duplicate of Reference 88.

3. Guedes PMM, de Andrade CM, Nunes DF, Pereira N, de Queiroga S, Machado- Coelho TBD. Inflammation enhances the risks of Stroke and Death in Chronic Chagas Disease patients. PLoS Negl Trop Dis. 2016;10:e0004669.	delete	3. Guedes PMM (2016): Duplicate of Reference 55.
4. Leon-Sarmiento FE, Mendoza E, Torres-Hillera M, Pinto N, Prada J, Silva CA, et al. Trypanosoma Cruzi-associated cerebrovascular disease: a case-control study in Eastern Colombia. J Neurol Sci. 2004;217:61–4.	delete	4. Leon-Sarmiento FE (2004): Duplicate of Reference 102.
5. de Melo ES, Bezerra R, de Holanda P, de Silva AC, Travassos MJL, Silva PP et al. TFL da,. Chagas disease stroke and associated risk factors: A case-control study. J Stroke Cerebrovasc Dis [Internet]. 2024 [cited 2024 Feb 21];33. https://www.strokejournal.org/article/S1052-3057(23)00485-8/abstract	delete	5. de Melo ES (2024): Duplicate of Reference 27.
6. Montanaro VVA, Hora TF, Da Silva CM, Santos CVDV, Lima MIR, Negrão EM, et al. Mortality and stroke recurrence in a Rehabilitation Cohort of patients with cerebral infarcts and Chagas Disease. Eur Neurol. 2018;79:177–84.	delete	6. Montanaro VVA (2018): Duplicate of Reference 65.
7. Montanaro VVA, da Silva CM, de Viana Santos CV, Lima MIR, Negrão EM, de Freitas GR. Ischemic stroke classification and risk of embolism in patients with Chagas disease. J Neurol. 2016;263:2411–5.	delete	7. Montanaro VVA (2016): Duplicate of Reference 62.
8. Montanaro VVA, Hora TF, da Silva CM, de Viana Santos CV, Lima MIR, de Jesus Oliveira EM, et al. Epidemiology of concurrent Chagas disease and ischemic stroke in a population attending a multicenter quaternary rehabilitation network in Brazil. Neurol Sci. 2019;40:2595–601.	delete	8. Montanaro VVA (2019): Duplicate of References 43.
9. Montanaro VVA, Hora TF, Guerra AA, Silva GS, Bezerra R, de P, Oliveira-Filho J, et al. Artificial Inteligence-based decision for the prediction of Cardioembolism in patients with Chagas Disease and ischemic stroke. J Stroke Cerebrovasc Dis off J Natl Stroke Assoc. 2021;30:106034.	delete	9. Montanaro VVA (2021): Duplicate of Reference 4.

10. Montanaro VVA, Silva GS, Oliveira Filho J, Pontes-Neto OM, do, Carmo Pereira Nunes M, de Bezerra R et al. P,. Exploring the Chagas disease-Stroke Connection: Findings from a Large Multicenter Study. Cerebrovasc Dis Basel Switz [Internet]. 2024; https://pubmed.ncbi.nlm.nih.gov/38228109/	delete	10. Montanaro VVA (2024): Duplicate of Reference 47.
11. Nunes MCP, Barbosa MM, Ribeiro ALP, Barbosa FBL, Rocha MOC. Ischemic cerebrovascular events in patients with Chagas cardiomyopathy: a prospective follow-up study. J Neurol Sci. 2009;278:96–101.	delete	11. Nunes MCP (2009): Duplicate of Reference 61.
12. Nunes MCP, Kreuser LJ, Ribeiro AL, Sousa GR, Costa HS, Botoni FA et al. Prevalence and risk factors of embolic cerebrovascular events Associated with Chagas Heart Disease. 2015;10:151.	delete	12. Nunes MCP (2015): Duplicate of Reference 75.
13. Oliveira-Filho J, Viana LC, Vieira-de-Melo RM, Faiçal F, Torreão JA, Villar FAGA, et al. Chagas Disease is an independent risk factor for stroke. Stroke. 2005;36:2015–7.	delete	13. Oliveira-Filho J (2005): Duplicate of Reference 24
14. Bestetti R. Stroke in a hospital-derived cohort of patients with chronic Chagas' disease. Acta Cardiol. 2000;55(1):33-8.	delete	14. Bestetti R (2000): Duplicate of Reference 87.
15. Carod-Artal FJ, Vargas AP, Horan TA, Nunes LG. Chagasic cardiomyopathy is independently associated with ischemic stroke in Chagas disease. Stroke. 2005;36(5):965-70.	delete	15. Carod-Artal FJ (2005): Duplicate of Reference 31.
16. Dias Junior JO, da Costa Rocha MO, de Souza AC, Kreuser LJ, de Souza Dias LA, Tan TC, et al. Assessment of the source of ischemic cerebrovascular events in patients with Chagas disease. Int J Cardiol. 2014;176(3):1352-4	delete	16. Dias Junior JO (2014): Duplicate of Reference 5.
17. Carod-Artal FJ, Vargas AP, Melo M, Horan TA. American trypanosomiasis (Chagas' disease): an unrecognised cause of stroke. J Neurol Neurosurg Psychiatry 2003;74:516-518.	delete	17. Carod-Artal FJ (2003): Duplicate of Reference 1.

Supplement 3 – Risk of Bias Assessment

3.1 Risk of bias assessment: (ROBINS-E)

CERQUEIRA et al., 2021. Chagas disease is an independente predictor of stroke and death in a cohort of heart failure patients.

**Domain 1: Risk of bias due to confounding**		
**Signalling questions**	**Response options**	**Comments**
1.1 Did the authors control for all the important confounding factors for which this was necessary?	Y	The model included relevant demographic, clinical, and echocardiographic variables.
1.2 **If Y/PY/WN to 1.1**: Were confounding factors that were controlled for (and for which control was necessary) measured validly and reliably by the variables available in this study?	Y	Standardized questionnaires, ECG, and Echocardiogram
1.3 **If Y/PY/WN to 1.1**: Did the authors control for any variables after the start of the exposure period being studied that could have been affected by the exposure?	Y	The authors adjusted the model for Atrial Fibrillation and Left Ventricular Ejection Fraction. In Chagas disease, AF and ventricular dysfunction are frequently mediators in the causal pathway to stroke and death, and not merely baseline confounding factors. Adjusting for mediators may cause overfitting bias. ECG and Echocardiogram
1.4 Did the use of negative controls, or other considerations, suggest serious uncontrolled confounding?	NA	
Risk of bias (due to confounding) in the estimated effect of exposure on the outcome	Some Concerns	Adjusting for variables that are in the causal pathway (such as Atrial Fibrillation and LVEF) may artificially attenuate the true effect of Chagas disease on stroke. However, since DC remained an independent predictor (HR 2.54) even with this overadjustment, the bias acts towards nullity (underestimating the risk), which strengthens the positive finding but methodologically raises modeling concerns.
Y = Yes; PY = Probably yes; PN = Probably no; N = No; SY = Strong yes; WY = Weak yes; SN = Strong no; WN = Weak no; NA = Not applicable; NI = No information		

Domain 2: Risk of bias arising from measurement of the exposure		
**Signalling questions**	**Response options**	**Comments**
2.1 Does the measured exposure well-characterize the exposure metric specified to be of interest in this study?	Y	
2.2 Was the exposure likely to be measured with error, or misclassified?	N	Duplicate ELISA tests are the gold standard.
2.3 If SY/WY to 2.2: Could mismeasurement or misclassification of exposure have been differential (i.e. related to the outcome or risk of the outcome)?	NA	
2.4 If SY/WY to 2.2 and N/PN/WY to 2.3: Is non-differential measurement error likely to bias the estimated effect of exposure on outcome?	NA	
Risk of bias (arising from measurement of exposure) in the estimated effect of exposure on the outcome	Low risk	Rigorous and centralized laboratory testing.
Y = Yes; PY = Probably yes; PN = Probably no; N = No; SY = Strong yes; WY = Weak yes; SN = Strong no; WN = Weak no; NA = Not applicable; NI = No information		

Domain 3: Risk of bias in selection of participants into the study (or into the analysis)		
**Signalling questions**	**Response options**	**Comments**
3.1 Did follow-up begin at (or close to) the start of the exposure window for most participants?	N	Patients already had established heart failure.
3.2 **If N/PN to 3.1**: Is the effect of exposure likely to be constant over the period of follow up analysed?	N	
3.3 Was selection of participants into the study (or into the analysis) based on participant characteristics observed after the start of the exposure window being studied? [*The exposure window is specified in D3*]	Y	Inclusion required a diagnosis of Heart Failure.
3.4 **If Y/PY to 3.3**: Were these characteristics likely to be influenced by exposure or a cause of exposure?	Y	A infecção por *T. cruzi* causa a Insuficiência Cardíaca
3.5 **If Y/PY to 3.4**: Were these characteristics likely to be influenced by the outcome or a cause of the outcome?	Y	Foram excluídos pacientes com histórico prévio de AVC).

3.6 **If N/PN to 3.2 or Y/PY to 3.5**: Is it likely that the analysis corrected for all of the potential selection biases identified in A and B above?	N	
3.7 **If N/PN to 3.2 or Y/PY to 3.5**: Did sensitivity analyses demonstrate that the likely impact of the potential selection biases identified in A or B above was minimal?	N	
Risk of bias (due to selection of participants into the study) in the estimated effect of exposure on the outcome	High risk	The study evaluates a cohort of "prevalent users" (patients who have already developed heart failure). This introduces a strong survival bias. Chagas disease patients who had a fatal or disabling stroke before developing symptomatic heart failure, or before arriving at the university outpatient clinic, were not included.
Y = Yes; PY = Probably yes; PN = Probably no; N = No; SN = Strong no; WN = Weak no; NA = Not applicable; NI = No information		

**Domain 4: Risk of bias due to post-exposure interventions**		
**Signalling questions**	**Response options**	**Comments**
4.1 Were there post-exposure interventions that were influenced by prior exposure during the follow-up period?	**PY**	The text states that "medications were adjusted according to heart failure symptoms" every 3-6 months.
4.2 **If Y/PY to 4.1**: Is it likely that the analysis corrected for the effect of post-exposure interventions that were influenced by prior exposure?	N	The multivariate model only used baseline aspirin use, without modeling time-dependent medications.
Risk of bias (due post-exposure interventions) in the estimated effect of exposure on the outcome	Some Concerns	Dynamic factors in treatment (e.g., introduction of anticoagulants upon development of AF during follow-up) do not appear to have been treated as time-dependent covariates in the Cox regression..
Y = Yes; PY = Probably yes; PN = Probably no; N = No; NA = Not applicable; NI = No information		
**Domain 5: Risk of bias due to missing data**		

**Signalling questions**	**Response options**	**Comments**
5.1 Were complete data on exposure status available for all, or nearly all, participants?	Y	Only 16 were excluded due to lack of serological tests.
5.2 Were complete data on the outcome available for all, or nearly all, participants?	**N**	Of the 565 patients recruited, 145 were excluded from the analysis due to loss to follow-up.
5.3 Were complete data on confounding variables available for all, or nearly all, participants?	Y	Multiple imputation was used for variables missing from the final models.
5.4 **If N/PN/NI to 5.1, 5.2 or 5.3**: Is the result based on a complete case analysis?	PY	
5.5 **If Y/PY/NI**: Was exclusion from the analysis because of missing data (in exposure, confounders or the outcome) likely to be related to the true value of the outcome?	NA	
5.6 **If N/PN to 5.5**: Were all or most predictors of missingness (in exposure, confounders or the outcome) included in the analysis model?	NA	
5.7 **If N/PN to 5.4**: Was the analysis based on imputing missing values?	NA	
5.8 **If Y/PY to 5.7**: Was imputation performed appropriately?	NA	
5.9 **If N/PN to 5.7**: Was an appropriate alternative method used to correct for bias due to missing data?	NA	
5.10 **If PN/N/NI to 5.1, 5.2 or 5.3**: Is there evidence that the result was not biased by missing data?	PY	
Risk of bias (due to missing data) in the estimated effect of	High risk	The summary exclusion of 145 patients (25.6% of the original cohort) due to loss to

exposure on the outcome		follow-up presents a serious risk of attrition bias. Although the authors performed a sensitivity analysis using attrition as the dependent variable, the exclusion of 1/4 of the sample requires validation of the HR calculations.
Y = Yes; PY = Probably yes; PN = Probably no; N = No; SY = Strong yes; WY = Weak yes; NA = Not applicable; NI = No information		
**Domain 6: Risk of bias arising from measurement of the outcome**		
**Signalling questions**	**Response options**	**Comments**
6.1 Could measurement or ascertainment of the outcome have differed between exposure groups or levels of exposure?	N	CT/MRI for stroke;.
6.2 Were outcome assessors aware of study participants' exposure history?	N	There was active searching by phone and a request for neuroimaging.
6.3 **If Y/PY/NI to 6.2**: Could assessment of the outcome have been influenced by knowledge of participants' exposure history?	NA	
Risk of bias (arising from measurement of outcomes) in the estimated effect of exposure on the outcome	Low risk	Strict diagnostic criteria for the outcome.
Y = Yes; PY = Probably yes; PN = Probably no; N = No; SY = Strong yes; WY = Weak yes; NA = Not applicable; NI = No information		
**Domain 7: Risk of bias in selection of the reported result**		
**Signalling questions**	**Response options**	**Comments**
7.1 Was the result reported in accordance with an available, pre-determined analysis plan?	Y	
7.2 **If N/PN/NI to 7.1**: Is the reported effect estimate likely to be selected, based on desirability of the magnitude (or statistical significance) of the estimated effect of exposure	NA	

on outcome, from multiple *exposure measurements* within the exposure domain?		
7.3 Is the reported effect estimate likely to be selected, based on desirability of the magnitude (or statistical significance) of the estimated effect of exposure on outcome, from multiple *outcome measurements* within the outcome domain?	N	
7.4 Is the reported effect estimate likely to be selected, based on desirability of the magnitude (or statistical significance) of the estimated effect of exposure on outcome, from multiple *analyses* of the exposure-outcome relationship?	N	
7.5 Is the reported effect estimate likely to be selected, based on the basis of desirability of the results (e.g. statistical significance), from different *subgroups*?	N	
Risk of bias (due to selection of the reported result) in the estimated effect of exposure on the outcome	Low risk	Transparent reporting of statistical models.
Y = Yes; PY = Probably yes; PN = Probably no; N = No; NA = Not applicable; NI = No information		

Overall risk of bias

	Response options	Comments
Overall risk of bias	High risk	Survival bias inherent in the exclusive inclusion of patients who have already reached the stage of Heart Failure and have not had a previous stroke.<br><br>Massive loss to follow-up, with exclusion of more than 25% of the initial cohort (145 of 565 patients) before the main analysis, generating a strong attrition bias. Additionally, there are concerns (Domain 1) regarding statistical overfitting due to the

		inclusion of mediating variables (AF and LVEF) in the Cox models.

This work is licensed under a Creative Commons Attribution-NonCommercial-NoDerivatives 4.0 International License.

Risk of bias assessment:

LIMA-COSTA et al., 2010. Chagas Disease Predicts 10-Year Stroke Mortality in Community-Dwelling Elderly The Bambuí Cohort Study of Aging

**Domain 1: Risk of bias due to confounding**		
**Signalling questions**	**Response options**	**Comments**
1.1 Did the authors control for all the important confounding factors for which this was necessary?	Y	Model 3 in Table 2 adjusts for age, sex, education, conventional CV risk factors, and CRP.
1.2 **If Y/PY/WN to 1.1**: Were confounding factors that were controlled for (and for which control was necessary) measured validly and reliably by the variables available in this study?	Y	Standardized interviews, fasting blood tests, ECGs read by a specialized center.

Domain 1: Risk of bias due to confounding		
**Signalling questions**	**Response options**	**Comments**
1.3 **If Y/PY/WN to 1.1**: Did the authors control for any variables after the start of the exposure period being studied that could have been affected by the exposure?	N	No.
1.4 Did the use of negative controls, or other considerations, suggest serious uncontrolled confounding?	NA	No.
Risk of bias (due to confounding) in the estimated effect of exposure on the outcome	Low risk	
Y = Yes; PY = Probably yes; PN = Probably no; N = No; SY = Strong yes; WY = Weak yes; SN = Strong no; WN = Weak no; NA = Not applicable; NI = No information		

**Domain 2: Risk of bias arising from measurement of the exposure**		
**Signalling questions**	**Response options**	**Comments**
2.1 Does the measured exposure well-characterize the exposure metric specified to be of interest in this study?	Y	Chronic infection
2.2 Was the exposure likely to be measured with error, or misclassified?	N	Three concordant serological tests were required; discordant tests were excluded. Kappa concordance of 0.989
2.3 If SY/WY to 2.2: Could mismeasurement or misclassification of exposure have been differential (i.e. related to the outcome or risk of the outcome)?	NA	
2.4 If SY/WY to 2.2 and N/PN/WY to 2.3: Is non-differential measurement error likely to bias the estimated effect of exposure on outcome?	NA	
Risk of bias (arising from measurement of exposure) in the estimated effect of exposure on the outcome	Low risk	Triple laboratory testing ensures very high internal validity for determining exposure.
Y = Yes; PY = Probably yes; PN = Probably no; N = No; SY = Strong yes; WY = Weak yes; SN = Strong no; WN = Weak no; NA = Not applicable; NI = No information		

**Domain 3: Risk of bias in selection of participants into the study (or into the analysis)**		
**Signalling questions**	**Response options**	**Comments**
3.1 Did follow-up begin at (or close to) the start of the exposure window for most participants?	N	The infection occurred in youth, but the cohort only included individuals who reached 60 years of age or older in 1997.
3.2 **If N/PN to 3.1**: Is the effect of exposure likely to be constant over the period of follow up analysed?	N	The risk of death from Chagas disease is cumulative.
3.3 Was selection of participants into the study (or into the analysis) based on participant characteristics observed after the start of the exposure window being studied? [*The exposure window is specified in D3*]	Y	Survival up to 60 years of age..

3.4 **<u>If Y/PY to 3.3</u>**: Were these characteristics likely to be influenced by exposure or a cause of exposure?	Y	A Doença de Chagas causa mortalidade prematura por insuficiência cardíaca e morte súbita antes dos 60 anos).
3.5 **<u>If Y/PY to 3.4</u>**: Were these characteristics likely to be influenced by the outcome or a cause of the outcome?	N	
3.6 **<u>If N/PN to 3.2 or Y/PY to 3.5</u>**: Is it likely that the analysis corrected for all of the potential selection biases identified in A and B above?	N	
3.7 **<u>If N/PN to 3.2 or Y/PY to 3.5</u>**: Did sensitivity analyses demonstrate that the likely impact of the potential selection biases identified in A or B above was minimal?	N	
Risk of bias (due to selection of participants into the study) in the estimated effect of exposure on the outcome	High risk	Este é um viés clássico de estudos de coorte com usuários prevalentes (*Prevalent User Bias / Survivor Bias*). Como a Doença de Chagas mata muitos pacientes antes dos 60 anos, a amostra do estudo é composta apenas por "sobreviventes resilientes" à infecção. Isso tende a subestimar a verdadeira força da associação entre Chagas e AVC na população geral, pois os casos mais graves já foram a óbito antes de entrar na coorte.
Y = Yes; PY = Probably yes; PN = Probably no; N = No; SN = Strong no; WN = Weak no; NA = Not applicable; NI = No information		

**Domain 4: Risk of bias due to post-exposure interventions**		
**Signalling questions**	**Response options**	**Comments**
4.1 Were there post-exposure interventions that were influenced by prior exposure during the follow-up period?	<u>N</u>	The text explicitly states: "None of the cohort participants had a history of antitrypanosomal medication... and none received treatment during the period").
4.2 **If Y/PY to 4.1**: Is it likely that the analysis corrected for the effect of post-exposure interventions that were influenced by prior exposure?	NA	
Risk of bias (due post-exposure interventions) in the	Low risk	The absence of trypanocidal treatment eliminates the bias toward specific

estimated effect of exposure on the outcome		intervention. The use of digoxin reflects the management of consequences (heart failure), which is expected in the natural history of the disease.
Y = Yes; PY = Probably yes; PN = Probably no; N = No; NA = Not applicable; NI = No information		
**Domain 5: Risk of bias due to missing data**		
**Signalling questions**	**Response options**	**Comments**
5.1 Were complete data on exposure status available for all, or nearly all, participants?	<u>Y</u>	1398 included out of 1606 eligible
5.2 Were complete data on the outcome available for all, or nearly all, participants?	<u>Y</u>	Only 6% loss to follow-up in 10 years.
5.3 Were complete data on confounding variables available for all, or nearly all, participants?	<u>Y</u>	
5.4 **<u>If N/PN/NI to 5.1, 5.2 or 5.3</u>**: Is the result based on a complete case analysis?	NA	
5.5 **<u>If Y/PY/NI</u>**: Was exclusion from the analysis because of missing data (in exposure, confounders or the outcome) likely to be related to the true value of the outcome?	NA	
5.6 **If N/PN to 5.5**: Were all or most predictors of missingness (in exposure, confounders or the outcome) included in the analysis model?	NA	
5.7 **If N/PN to 5.4**: Was the analysis based on imputing missing values?	NA	
5.8 **If Y/PY to 5.7**: Was imputation performed appropriately?	NA	
5.9 **If N/PN to 5.7**: Was an appropriate alternative method used to correct for bias due to missing data?	NA	

5.10 **<u>If PN/N/NI to 5.1, 5.2 or 5.3</u>**: Is there evidence that the result was not biased by missing data?	NA	
Risk of bias (due to missing data) in the estimated effect of exposure on the outcome	Low risk	Very high cohort retention rate (94%) for a 10-year follow-up in elderly individuals.
Y = Yes; PY = Probably yes; PN = Probably no; N = No; SY = Strong yes; WY = Weak yes; NA = Not applicable; NI = No information		
**Domain 6: Risk of bias arising from measurement of the outcome**		
**Signalling questions**	**Response options**	**Comments**
6.1 Could measurement or ascertainment of the outcome have differed between exposure groups or levels of exposure?	<u>N</u>	Death registered in the official system.
6.2 Were outcome assessors aware of study participants' exposure history?	<u>N</u>	The authors performed a robust sensitivity analysis, varying the accuracy of the death certificate between 60% and 80%, and the statistical association remained significant.
6.3 **<u>If Y/PY/NI to 6.2</u>**: Could assessment of the outcome have been influenced by knowledge of participants' exposure history?	NA	
Risk of bias (arising from measurement of outcomes) in the estimated effect of exposure on the outcome	Low risk	The sensitivity analysis for misclassification errors in death certificates demonstrates great methodological rigor.
Y = Yes; PY = Probably yes; PN = Probably no; N = No; SY = Strong yes; WY = Weak yes; NA = Not applicable; NI = No information		
**Domain 7: Risk of bias in selection of the reported result**		
**Signalling questions**	**Response options**	**Comments**
7.1 Was the result reported in accordance with an available, pre-determined analysis plan?	<u>Y</u>	

7.2 **If N/PN/NI to 7.1**: Is the reported effect estimate likely to be selected, based on desirability of the magnitude (or statistical significance) of the estimated effect of exposure on outcome, from multiple *exposure measurements* within the exposure domain?	NA	
7.3 Is the reported effect estimate likely to be selected, based on desirability of the magnitude (or statistical significance) of the estimated effect of exposure on outcome, from multiple *outcome measurements* within the outcome domain?	N	
7.4 Is the reported effect estimate likely to be selected, based on desirability of the magnitude (or statistical significance) of the estimated effect of exposure on outcome, from multiple *analyses* of the exposure-outcome relationship?	N	
7.5 Is the reported effect estimate likely to be selected, based on the basis of desirability of the results (e.g. statistical significance), from different *subgroups*?	N	
Risk of bias (due to selection of the reported result) in the estimated effect of exposure on the outcome	Low risk	Relato transparente e alinhado com os objetivos do estudo de coorte.
Y = Yes; PY = Probably yes; PN = Probably no; N = No; NA = Not applicable; NI = No information		

Overall risk of bias

	**Response options**	**Comments**
Overall risk of bias	High risk	The Bambuí Cohort study is methodologically brilliant in its execution, exposure assessment (D2), loss control (D5), and statistical analysis (D1 and D6). However, due to the nature of the ROBINS-E instrument, the study receives a High Risk

		classification in Domain 3 (Participant Selection). Because it is a cohort of elderly individuals (≥ 60 years) evaluating an infection acquired in youth, there is a strong survival bias. Individuals most susceptible to cardiovascular damage from T. cruzi likely died before age 60 and were not included in the study. The direction of this bias suggests that the Hazard Ratio of 2.35 found for stroke mortality is, in fact, a conservative (underestimated) estimate of the true lifetime risk posed by Chagas disease.

This work is licensed under a [Creative Commons Attribution-NonCommercial-NoDerivatives 4.0 International License](#).

Risk of bias assessment:

MONTANARO et al., 2019 Cerebral infarct topography of atrial fibrillation and Chagas disease

Domain 1: Risk of bias due to confounding		
**Signalling questions**	**Response options**	**Comments**
1.1 Did the authors control for all the important confounding factors for which this was necessary?	WN	The text explicitly states: "The patients were not matched for age and sex." Furthermore, although risk factors were measured, the results show striking differences in statin and anticoagulant use between the groups that do not appear to have been adjusted for in multivariate models of mortality and mRS outcomes.
1.2 **If Y/PY/WN to 1.1**: Were confounding factors that were controlled for (and for which control was necessary) measured validly and reliably by the variables available in this study?	Y	They were extracted from standardized electronic rehabilitation records.
1.3 **If Y/PY/WN to 1.1**: Did the authors control for any variables after the start of the exposure period being studied that could have been affected by the exposure?	NA	
1.4 Did the use of negative controls, or other considerations, suggest serious uncontrolled confounding?	NA	
Risk of bias (due to confounding) in the estimated effect of exposure on the outcome	High risk	The text reveals significant baseline imbalances (e.g., statin use 82% vs. 51%; anticoagulant use 50% vs. 0%). Since the study is only comparative (Chi-square/Kaplan-Meier) and does not mention multivariate regression to adjust for these differences in prior treatment, the comparison of mortality and severity (mRS) is highly susceptible to confounding bias.
Y = Yes; PY = Probably yes; PN = Probably no; N = No; SY = Strong yes; WY = Weak yes; SN = Strong no; WN = Weak no; NA = Not applicable; NI = No information		

**Domain 2: Risk of bias arising from measurement of the exposure**		
**Signalling questions**	**Response options**	**Comments**
2.1 Does the measured exposure well-characterize the exposure metric specified to be of interest in this study?	Y	Diagnoses confirmed by dual serology for Crohn's disease and ECG/Holter for atrial fibrillation.
2.2 Was the exposure likely to be measured with error, or misclassified?	N	The battery of tests described [TTE, ETE, Holter, Doppler] and the classification by two independent neurologists with a Kappa index of 0.909 indicate very high accuracy.
2.3 If SY/WY to 2.2: Could mismeasurement or misclassification of exposure have been differential (i.e. related to the outcome or risk of the outcome)?	NA	
2.4 If SY/WY to 2.2 and N/PN/WY to 2.3: Is non-differential measurement error likely to bias the estimated effect of exposure on outcome?	NA	
Risk of bias (arising from measurement of exposure) in the estimated effect of exposure on the outcome	Low risk	The methodology for defining the etiology (exposure) is extremely rigorous, using the Harvard CCS system and consensus among blinded experts.
Y = Yes; PY = Probably yes; PN = Probably no; N = No; SY = Strong yes; WY = Weak yes; SN = Strong no; WN = Weak no; NA = Not applicable; NI = No information		

**Domain 3: Risk of bias in selection of participants into the study (or into the analysis)**		
**Signalling questions**	**Response options**	**Comments**
3.1 Did follow-up begin at (or close to) the start of the exposure window for most participants?	N	Retrospective study of patients already with sequelae.
3.2 **If N/PN to 3.1**: Is the effect of exposure likely to be constant over the period of follow up analysed?	PY	
3.3 Was selection of participants into the study (or into the analysis) based on participant characteristics observed after the start of the exposure window being studied? [*The*	Y	Inclusion required admission to a neurological rehabilitation service.

exposure window is specified in D3]		
3.4 **<u>If Y/PY to 3.3</u>**: Were these characteristics likely to be influenced by exposure or a cause of exposure?	<u>PY</u>	
3.5 **<u>If Y/PY to 3.4</u>**: Were these characteristics likely to be influenced by the outcome or a cause of the outcome?	Y	To qualify for rehabilitation, the patient must have survived the acute phase of the stroke and have a degree of morbidity that requires rehabilitation, but not so severe as to be bedridden in a persistent vegetative state with no potential for recovery.
3.6 **<u>If N/PN to 3.2 or Y/PY to 3.5</u>**: Is it likely that the analysis corrected for all of the potential selection biases identified in A and B above?	N	
3.7 **<u>If N/PN to 3.2 or Y/PY to 3.5</u>**: Did sensitivity analyses demonstrate that the likely impact of the potential selection biases identified in A or B above was minimal?	NA	
Risk of bias (due to selection of participants into the study) in the estimated effect of exposure on the outcome	High risk	The text states: "All patients who were admitted to the neurological rehabilitation service... were included." The average time from stroke to admission was 2.5 to 3 years. This constitutes a massive survival bias (Neyman bias). The patients analyzed represent only long-term survivors who had access to a center of excellence in rehabilitation, not reflecting the actual mortality or morbidity of the acute phase of the disease in the general population.
Y = Yes; PY = Probably yes; PN = Probably no; N = No; SN = Strong no; WN = Weak no; NA = Not applicable; NI = No information		

**Domain 4: Risk of bias due to post-exposure interventions**		
**Signalling questions**	**Response options**	**Comments**
4.1 Were there post-exposure interventions that were influenced by prior exposure during the follow-up period?	Y	Patients with atrial fibrillation (AF) or cardioembolic cardiac arrest (CA) received more anticoagulants and statins than the CA-indeterminate group.
4.2 **If Y/PY to 4.1**: Is it likely that the analysis corrected for the effect of post-exposure interventions that were	NI	There is no mention of statistical adjustment for medication use in the survival curves.

influenced by prior exposure?		
Risk of bias (due post-exposure interventions) in the estimated effect of exposure on the outcome	High risk	The drastic differences in clinical management (statins and anticoagulants) documented in the results directly affect the mortality and recurrence outcomes analyzed in the study.
Y = Yes; PY = Probably yes; PN = Probably no; N = No; NA = Not applicable; NI = No information		
**Domain 5: Risk of bias due to missing data**		
**Signalling questions**	**Response options**	**Comments**
5.1 Were complete data on exposure status available for all, or nearly all, participants?	Y	
5.2 Were complete data on the outcome available for all, or nearly all, participants?	PY	The text mentions that 20% of the DC group missed their last appointment, but that phone contact was made to determine mortality/recurrence rates.
5.3 Were complete data on confounding variables available for all, or nearly all, participants?	Y	admission routine
5.4 **If N/PN/NI to 5.1, 5.2 or 5.3**: Is the result based on a complete case analysis?	NA	
5.5 **If Y/PY/NI**: Was exclusion from the analysis because of missing data (in exposure, confounders or the outcome) likely to be related to the true value of the outcome?	NA	
5.6 **If N/PN to 5.5**: Were all or most predictors of missingness (in exposure, confounders or the outcome) included in the analysis model?	NA	
5.7 **If N/PN to 5.4**: Was the analysis based on imputing missing values?	NA	
5.8 **If Y/PY to 5.7**: Was imputation performed appropriately?	NA	

5.9 **If N/PN to 5.7**: Was an appropriate alternative method used to correct for bias due to missing data?	NA	
5.10 **<u>If PN/N/NI to 5.1, 5.2 or 5.3</u>**: Is there evidence that the result was not biased by missing data?	NA	
Risk of bias (due to missing data) in the estimated effect of exposure on the outcome	Low risk	The authors describe an active effort (telephone contact) to mitigate loss to follow-up (attrition bias) for mortality and recurrence outcomes.
Y = Yes; PY = Probably yes; PN = Probably no; N = No; SY = Strong yes; WY = Weak yes; NA = Not applicable; NI = No information		
**Domain 6: Risk of bias arising from measurement of the outcome**		
**Signalling questions**	**Response options**	**Comments**
6.1 Could measurement or ascertainment of the outcome have differed between exposure groups or levels of exposure?	<u>N</u>	Neuroimaging for topography; death for mortality.
6.2 Were outcome assessors aware of study participants' exposure history?	<u>N</u>	For the topography analysis, two independent neurologists, blinded to the etiology, evaluated the images.
6.3 **<u>If Y/PY/NI to 6.2</u>**: Could assessment of the outcome have been influenced by knowledge of participants' exposure history?	NA	
Risk of bias (arising from measurement of outcomes) in the estimated effect of exposure on the outcome	Low risk	Blinding the evaluators of CT/MRI images regarding Chagas serology and etiology (FA) is a very strong point of the study, eliminating measurement bias for the topographic outcome.
Y = Yes; PY = Probably yes; PN = Probably no; N = No; SY = Strong yes; WY = Weak yes; NA = Not applicable; NI = No information		
**Domain 7: Risk of bias in selection of the reported result**		
**Signalling questions**	**Response options**	**Comments**

7.1 Was the result reported in accordance with an available, pre-determined analysis plan?	Y	
7.2 **If N/PN/NI to 7.1**: Is the reported effect estimate likely to be selected, based on desirability of the magnitude (or statistical significance) of the estimated effect of exposure on outcome, from multiple *exposure measurements* within the exposure domain?	NA	
7.3 Is the reported effect estimate likely to be selected, based on desirability of the magnitude (or statistical significance) of the estimated effect of exposure on outcome, from multiple *outcome measurements* within the outcome domain?	N	
7.4 Is the reported effect estimate likely to be selected, based on desirability of the magnitude (or statistical significance) of the estimated effect of exposure on outcome, from multiple *analyses* of the exposure-outcome relationship?	N	
7.5 Is the reported effect estimate likely to be selected, based on the basis of desirability of the results (e.g. statistical significance), from different *subgroups*?	N	
Risk of bias (due to selection of the reported result) in the estimated effect of exposure on the outcome	Low risk	The reported results directly correspond to the objectives and methodology described, with no indication of statistical data selection.
Y = Yes; PY = Probably yes; PN = Probably no; N = No; NA = Not applicable; NI = No information		

Overall risk of bias

	**Response options**	**Comments**
Overall risk of bias	High risk	Although the classification of exposure (Domain 2) and the blinded assessment of

		stroke topography (Domain 6) are methodologically excellent, the study suffers from serious structural flaws inherent in its retrospective design in a rehabilitation center. Domain 3 (Selection) presents a High Risk due to massive survival bias (mean time of 3 years between stroke and admission to rehabilitation). Furthermore, Domains 1 (Confusion) and 4 (Post-exposure interventions) present a High Risk, as the results show severe imbalances in the use of statins and anticoagulants between the groups, with no evidence that mortality and morbidity analyses (mRS) have been statistically adjusted for these crucial therapeutic differences.

This work is licensed under a Creative Commons Attribution-NonCommercial-NoDerivatives 4.0 International License.

3.1.1 Supplementary figure 1: Risk of bias assessment of cohort and case–control studies according to the ROBINS-E tool.

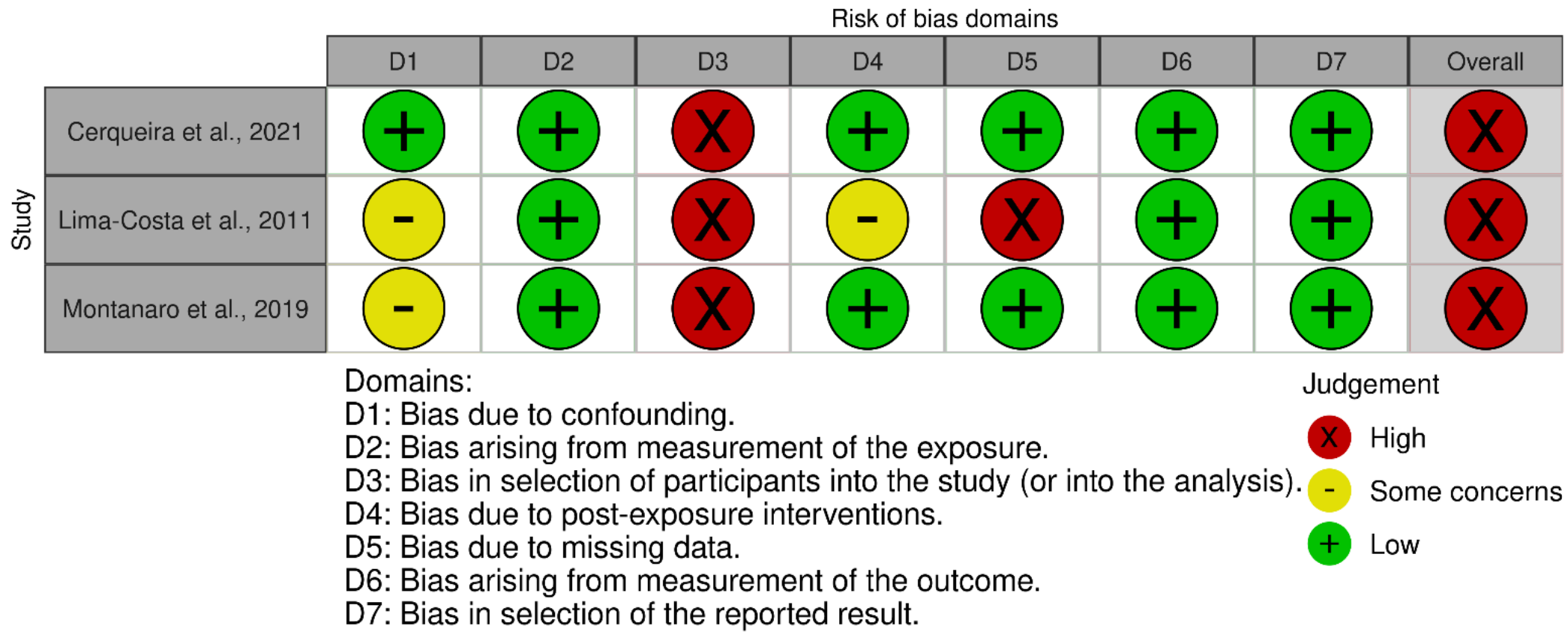

3.2 Assessment of the methodological quality of the included cross-sectional studies according to the JBI instrument.

**Author (Year)**	**Q1. . Were the criteria for inclusion in the sample clearly defined?**	**Q2. Were the study subjects and the setting described in detail?**	**Q3. Was the exposure measured in a valid and reliable way?**	**Q4. Were objective, standard criteria used for measurement of the condition?**	**Q5. Were confounding factors identified?**	**Q6. Were strategies to deal with confounding factors stated?**	**Q7. Were the outcomes measured in a valid and reliable way?**	**Q8. Was appropriate statistical analysis used?**	**Methodological Quality**
Matta et al., 2012	Y	Y	Y	Y	Y	Y	Y	Y	High
Jesus et al., 2011	Y	Y	U	Y	Y	Y	Y	Y	Moderate
Dias et al., 2009	Y	Y	Y	Y	Y	Y	Y	Y	High
Oliveira-filho et al., 2005	Y	Y	Y	Y	Y	Y	Y	Y	High

Legend – Y=Yes, N=No, U=Unclear, NA=Not applicable.

3.2.1 Supplementary figure 2: Methodological quality assessment of cross-sectional studies according to the JBI tool.

Study	Q1	Q2	Q3	Q4	Q5	Q6	Q7	Q8	Overall
Matta et al., 2012	+	+	+	+	+	+	+	+	+
Jesus et al., 2011	+	+	~	+	+	+	+	+	~
Dias et al., 2009	+	+	+	+	+	+	+	+	+
Oliveira-Filho et al, 2005	+	+	+	+	+	+	+	+	+

(+) High (~) Moderate (−) Low

Supplement 4 – Scripts

```
# Load the packages
library(meta)


# Figure 3: Cohort studies
dat_coort <- data.frame(study_name = c("Cerqueira et al., 2021",
                                       "Lima Costa et al., 2010"),
                        events_trat = c(16, 25),
                        n_trat = c(210, 524),
                        event_ctl = c(9, 20),
                        n_ctl = c(194, 874))
m_coort <- metabin(event.e = events_trat, n.e = n_trat,
                   event.c = event_ctl, n.c = n_ctl,
                   studlab = study_name, common = FALSE,
                   label.e = "With ChD", label.c = "Without ChD",
                   data = dat_coort)
summary(m_coort)
forest(m_coort,
       smlab = "Relative Risk",
       leftlabs = c("Study"),
       rightlabs = c("RR", "95% CI"))
#
png(file = "Forest_coort.png", width = 2600, height = 1200, res = 300)
forest(m_coort,
       smlab = "Relative Risk",
       leftlabs = c("Study"),
       rightlabs = c("RR", "95% CI"))
dev.off()
# Figure 4: Cross-sectional studies
dat_trans <- data.frame(study_name = c("Matta et al., 2012",
                                       "Dias et al., 2009",
```

```
                        "Jesus et al., 2011",
                        "Oliveira Filho et al., 2005"),
                 events_trat = c(57, 6, 7, 22),
                 n_trat = c(329, 37, 62, 147),
                 event_ctl = c(51, 6, 6, 10),
                 n_ctl = c(461, 42, 82, 158),
                 Complication = c("Chagas cardiomyopathy",
                                  "Chagas cardiomyopathy",
                                  "Congestive Heart Failure",
                                  "Chagas cardiomyopathy"))
m_trans <- metabin(event.e = events_trat, n.e = n_trat,
               event.c = event_ctl, n.c = n_ctl,
               studlab = study_name, common = FALSE,
               subgroup = Complication,
               label.e = "With ChD", label.c = "Without ChD",
               data = dat_trans)
summary(m_trans)
forest(m_trans,
       smlab = "Prevalence Ratio",
       leftlabs = c("Study"),
       rightcols = c("effect", "ci", "w.random"),
       rightlabs = c("PR", "95% CI", "Weight"))
#
png(file = "Forest_trans.png", width = 2600, height = 1200, res = 300)
forest(m_trans,
       smlab = "Prevalence Ratio",
       leftlabs = c("Study"),
       rightcols = c("effect", "ci", "w.random"),
       rightlabs = c("PR", "95% CI", "Weight"))
dev.off()
```

Supplement 5 – Database

Santos AC. Dados de replicação para: Associação entre doença de Chagas e ocorrência de Acidente Vascular Cerebral: Revisão sistemática com metanálise [Internet]. SciELO Data; 2026. Disponível em: https://doi.org/10.48331/SCIELODATA.IOXGSK doi:10.48331/SCIELODATA.IOXGSK
